# Integrative Single-Cell and Bulk Transcriptomic Analyses with Spatial Validation Identify a Residual Fatty Acid–EMT Subset Driving Chemotherapy Resistance in Triple-Negative Breast Cancer via MIF- and MK-Mediated Ligand–Receptor Signaling

**DOI:** 10.3390/ijms27146157

**Published:** 2026-07-09

**Authors:** Zinab O. Doha, Renad R. Alharbi, Mohrah S. Aljohani, Haneen M. Alharbi, Hakeemah H. Alnakhle, Ghadi S. Alharbi, Shatha A. Alerwi

**Affiliations:** 1 Department of Clinical Laboratory Sciences, College of Applied Medical Sciences, Taibah University, Madinah 42353, Saudi Arabia; 2 Health and Life Research Center, Taibah University, Madinah 42353, Saudi Arabia

**Keywords:** triple-negative breast cancer, chemotherapy resistance, fatty acid–EMT co-expression, single-cell RNA sequencing, tumor microenvironment, MIF–CD74–CXCR4, MDK–NCL, T-cell exhaustion, spatial proteomics

## Abstract

Chemotherapy resistance in triple-negative breast cancer (TNBC) remains a critical clinical challenge, with a substantial proportion of patients failing to achieve pathological complete response following neoadjuvant chemotherapy (NAC). Using an integrative single-cell RNA sequencing (scRNA-seq), bulk transcriptomic, and spatial proteomic framework, we aimed to identify the malignant epithelial subset driving this resistance and the intercellular signaling axes through which it reprograms the tumor microenvironment (TME). scRNA-seq analysis of NAC-treated breast tumors revealed a Fatty Acid–EMT co-expressing epithelial subset (FA-EMT) that is selectively enriched in the chemotherapy-resistant residuum. Critically, FA-EMT co-expression—rather than either program individually—most powerfully predicted chemotherapy resistance and reduced overall survival across two independent bulk transcriptomic cohorts comprising 277 TNBC patients (*p* < 0.001). CellChat ligand–receptor analysis established FA-EMT cells as the dominant TME signaling hub, deploying MDK–NCL and MIF–CD74–CXCR4 axes to simultaneously suppress adaptive and innate anti-tumor immunity via T-cell exhaustion, Treg activation, and the expansion of myeloid-derived suppressor cells. Spatial CyCIF validation in a published paclitaxel-resistant TNBC mouse model (n = 69 cores) confirmed significant Metabolic-EMT enrichment in resistant tumor cores (*p* = 0.0085) with physical co-localization with immunosuppressive MDSC and Treg populations. These findings establish the FA-EMT subset as a key cellular driver of treatment failure in TNBC and nominate MDK–NCL and MIF–CD74–CXCR4 as mechanistically grounded therapeutic targets with the potential to dismantle the FA-EMT-driven immunosuppressive niche and sensitize chemotherapy-resistant TNBC to cytotoxic treatment.

## 1. Introduction

Breast cancer remains the most frequently diagnosed malignancy and the leading cause of cancer-related mortality in women worldwide, with an estimated 2.3 million new cases and over 685,000 deaths recorded annually [1]. Among its molecular subtypes, triple-negative breast cancer (TNBC)—defined by the absence of estrogen receptor, progesterone receptor, and human epidermal growth factor receptor 2 (HER2) expression—represents 15–20% of all breast cancer cases yet accounts for a disproportionate share of mortality, particularly within the first five years of diagnosis [2,3]. Because TNBC lacks the targetable receptors that drive hormonal and HER2-directed therapies, neoadjuvant chemotherapy (NAC) remains the cornerstone of treatment. However, a substantial proportion of patients fail to achieve pathological complete response (pCR), and those with residual disease face markedly worse recurrence-free and overall survival outcomes [4,5]. The biological determinants of this chemotherapy resistance remain incompletely understood, representing one of the most pressing unresolved challenges in breast oncology.

Emerging evidence indicates that chemotherapy resistance in TNBC is not a uniform cellular phenomenon but is instead driven by transcriptionally distinct subpopulations of malignant cells that persist and expand under therapeutic pressure [6,7]. These resistant cell subsets not only survive cytotoxic pressure but also drive dynamic remodeling of the immune and stromal compartments, collectively establishing an immunosuppressive niche that further shields them from elimination [8,9]. However, the precise cellular and molecular mechanisms through which residual malignant epithelial cells actively orchestrate this immunosuppressive remodeling—rather than merely coexisting within it—remain poorly defined. Identifying this mechanism is critical to defining actionable therapeutic targets and sensitizing TNBC to cytotoxic therapy.

The advent of single-cell RNA sequencing (scRNA-seq) has transformed our ability to resolve intratumoral heterogeneity at unprecedented resolution, enabling the identification of rare but functionally critical cell subpopulations that are obscured by conventional bulk transcriptomic approaches [10,11]. Complementary cell–cell communication frameworks, such as CellChat, now enable the systematic mapping of ligand–receptor interaction networks across the full cellular landscape of a tumor, offering mechanistic insight into how malignant cells communicate with and reprogram their microenvironment [12].

In this study, we leveraged a publicly available scRNA-seq atlas of chemotherapy-treated primary breast tumors (n = 5 NAC-treated patients) [13]—further validated using treatment-naïve samples from the same atlas across 19 patients (naïve TNBC vs. naïve non-TNBC) and 7 TNBC patients (chemo-treated vs naïve TNBC)—combined with two independent cohorts of bulk transcriptomic analysis, across 277 chemotherapy-treated TNBC patients [14,15], and spatial proteomic validation using published multiplexed protein imaging (CyCIF) data from a paclitaxel-resistant TNBC mouse model (n = 69 tumors) [16]. We identify a Fatty Acid–EMT co-expressing epithelial subset (FA-EMT) selectively enriched in the chemotherapy-resistant TNBC residuum and demonstrate that FA-EMT co-expression—rather than either program individually—most powerfully predicts chemotherapy resistance and reduced overall survival, establishing it as a functionally integrated resistance mechanism and a clinically actionable prognostic biomarker. Single-cell TME analysis reveals coordinated immunosuppressive remodeling encompassing T-cell exhaustion, Treg activation, cDC1 depletion, TAM and MDSC expansion, and iCAF dominance. Using CellChat ligand–receptor interaction analysis, we identify MDK–NCL and MIF–CD74–CXCR4 as the principal axes through which FA-EMT cells simultaneously suppress adaptive and innate anti-tumor immunity. Spatial CyCIF validation confirms significant Metabolic-EMT enrichment in resistant tumor cores with physical co-localization of immunosuppressive populations, providing direct tissue-level evidence for the resistance architecture. Together, these findings identify the FA-EMT subset as a key cellular driver of treatment failure in TNBC and nominate MDK–NCL and MIF–CD74–CXCR4 as mechanistically grounded therapeutic targets to sensitize chemotherapy-resistant TNBC to cytotoxic treatment.

## 2. Results

### 2.1. Single-Cell Profiling of TNBC Identifies a Residual Fatty Acid–EMT Co-Expressing Cancer Epithelial Subset Within a Myeloid-Enriched Immunosuppressive Microenvironment That Survives Chemotherapeutic Stress

**Figure 1:**
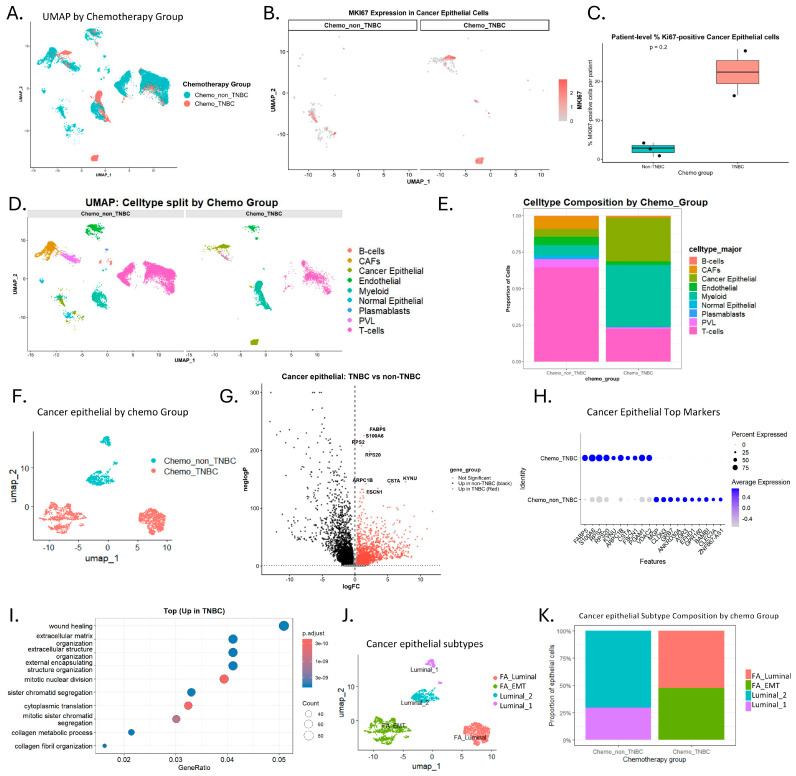
The post-chemotherapy TME is transcriptionally and compositionally distinct in TNBC, defined by a myeloid-enriched landscape and selective expansion of a co-proliferative FA-EMT epithelial subset. Single-cell RNA-seq data were derived from NAC-treated patients within a publicly available human breast cancer atlas [13]. (**A**) Integrated UMAP of all chemotherapy-treated single cells, colored by cohort (Chemo_non_TNBC, teal; Chemo_TNBC, red). (**B**) Split UMAP feature plots showing MKI67 expression in cancer epithelial cell Chemo_non_TNBC (left) and Chemo_TNBC (right) samples. Color intensity is proportional to the normalized expression level. (**C**) Boxplot of patient-level percentage of *MKI67*-positive cancer epithelial cells per chemotherapy group (Wilcoxon rank-sum test). (**D**) Split UMAPs colored by major cell lineage (B cells, CAFs, Cancer Epithelial, Endothelial, Myeloid, Normal Epithelial, Plasmablasts, PVL, T cells) as annotated by Wu et al. [13], shown separately for Chemo_non_TNBC and Chemo_TNBC cohorts. (**E**) Stacked bar plot of relative cell-type proportions within each chemotherapy group. (**F**) UMAP of the cancer epithelial sub-compartment, colored by chemotherapy group (Chemo_non_TNBC, green; Chemo_TNBC, orange/red). (**G**) Volcano plot of differential gene expression between Chemo_TNBC and Chemo_non_TNBC cancer epithelial cells (Wilcoxon rank-sum test, Benjamini–Hochberg FDR correction). Red dots: upregulated transcripts in Chemo_TNBC (log_2_FC > 0.5, adjusted *p* < 0.05). Black dots: downregulated transcripts. See also Supplementary Figure S1D–F and Supplementary Table S1. (**H**) Dot plot of the top differentially expressed marker genes across chemotherapy groups. Dot size: percentage of expressing cells; color intensity: scaled mean expression (blue, high; gray, low). (**I**) Dot plot of gene ontology (GO) biological process enrichment for Chemo_TNBC-upregulated genes (Benjamini–Hochberg correction). Dot size: gene count per term; color: adjusted *p*-value. (**J**) Annotated sub-cluster UMAP of the cancer epithelial compartment identifying four subtypes: FA-EMT (FA_EMT, green), FA-Luminal (FA_Luminal, salmon), Luminal_2 (teal), and Luminal_1 (purple). See Supplementary Figure S1I–K for marker definitions. (**K**) Stacked bar plot of cancer epithelial subtype proportions across chemotherapy groups.

To dissect the cellular architecture of the tumor microenvironment (TME) in TNBC versus non-TNBC following neoadjuvant chemotherapy (NAC), we leveraged a publicly available, high-resolution single-cell RNA sequencing (scRNA-seq) atlas of primary human breast tumors [13] and applied stringent clinical filtering to retain only NAC-treated patients. This yielded a study cohort of five patients stratified by molecular subtype: triple-negative breast cancer (Chemo_TNBC; n = 2) and non-triple-negative breast cancer (Chemo_non_TNBC; n = 3). Integrated UMAP dimensionality reduction in all chemotherapy-treated high-quality single cells (Figure 1A, and Supplementary Figure S1A,B) revealed a clear spatial separation between the two groups: Chemo_TNBC cells (red) were concentrated within discrete, transcriptionally restricted clusters, whereas Chemo_non_TNBC cells (teal) were broadly distributed across the UMAP landscape—a pattern reflective of greater phenotypic heterogeneity in the chemotherapy-responsive group relative to the transcriptionally constrained, resistant residuum.

Proliferative activity was assessed by overlaying *MKI67* expression onto UMAPs split by cohort (Figure 1B and Supplementary Figure S1C). In the Chemo_non_TNBC cohort, *MKI67*-positive cells were sparse and diffusely scattered across clusters, whereas the Chemo_TNBC cohort exhibited a dense, spatially localized concentration of cycling cells—indicating the persistence of an actively proliferating malignant population following cytotoxic treatment. Restricting quantification to the cancer epithelial compartment (Figure 1C), the median percentage of *MKI67*-positive cells in TNBC samples (~22%) was approximately seven-fold greater than in non-TNBC controls (~3%; *p* = 0.2). Although statistical significance was not reached owing to the limited patient numbers per group, the magnitude and directionality of this difference are biologically robust and consistent with chemotherapy-driven selection of a highly proliferative, treatment-resistant clonal population in TNBC. While formal pathological response classification was not uniformly available across all five NAC-treated patients in this atlas, the Chemo_TNBC group exhibited markedly higher *MKI67*-positive cancer epithelial cell proportions (~22% vs. ~3%), consistent with a more proliferative, treatment-refractory residuum. Accordingly, Chemo_TNBC and Chemo_non_TNBC are used throughout this study as molecular subtype comparators following NAC, with the TNBC group reflecting the transcriptional landscape associated with chemotherapy-resistant residual disease.

To resolve the lineage-level composition underlying these transcriptional differences, we visualized the nine major cell-type compartments previously annotated [9]—Cancer Epithelial, T cells, Myeloid cells, CAFs, Normal Epithelial, B cells, Plasmablasts, Endothelial, and PVL cells—and compared their distributions via split-UMAPs (Figure 1D) and relative proportion analysis (Figure 1E). The Chemo_non_TNBC landscape was architecturally diverse: T cells constituted the dominant population (>60% of total cells), accompanied by a rich stromal compartment including CAFs and PVL cells. In striking contrast, the Chemo_TNBC cohort was defined by disproportionate expansion of Cancer Epithelial and Myeloid populations, alongside profound depletion of T cells and stromal diversity. These compositional differences—with T-cell and stromal dominance in Chemo_non_TNBC and Cancer Epithelial and Myeloid expansion in Chemo_TNBC—likely reflect both intrinsic molecular subtype biology and chemotherapy-associated microenvironmental remodeling. While the patient number is limited (n = 5), the dataset encompasses 7373 high-quality single cells from Chemo_TNBC and 13,287 from Chemo_non_TNBC, providing sufficient cellular resolution to characterize the transcriptional landscape of each compartment at single-cell depth. These observations should therefore be interpreted as hypothesis-generating rather than definitive.

To define the transcriptional programs within the malignant epithelial compartment that drive TNBC chemotherapy resistance, we performed a focused sub-analysis restricted to cancer epithelial cells. UMAP (Figure 1F) demonstrated clear spatial separation between Chemo_TNBC (orange/red) and Chemo_non_TNBC (green) epithelial cells, confirming resistance-specific transcriptional reprogramming within the tumor cell fraction itself. Differential expression analysis (Figure 1G; Supplementary Figure S1D–F and Table S1) identified a Chemo_TNBC-enriched gene signature comprising the lipid-binding chaperone FABP5, the calcium-binding protein S100A6—a regulator of cellular proliferation, and the ribosomal component RPS2 (log_2_FC > 0.5; adjusted *p* < 0.05). Dot plot validation (Figure 1H) confirmed that these markers exhibited both higher mean expression and greater detection frequency in Chemo_TNBC relative to Chemo_non_TNBC cells. The concurrent upregulation of FABP5—a lipid-binding chaperone central to fatty acid uptake and utilization—alongside the EMT-proliferative marker S100A6 and the cell cycle regulators *MKI67* (Figure 1C) implicates a coordinated molecular strategy in which TNBC epithelial cells simultaneously engage lipid-based metabolic adaptation, EMT, and active cell-cycle progression to survive chemotherapeutic stress. Gene ontology enrichment analysis of the Chemo_TNBC-upregulated signature (Figure 1I; Supplementary Figure S1D) revealed significant enrichment of extracellular matrix (ECM) organization, extracellular structure organization, cytoplasmic translation, and collagen metabolic process pathways—indicating the activation of a mesenchymally primed, ECM-remodeling transcriptional program in chemorefractory TNBC epithelial cells, consistent with hallmarks of epithelial–mesenchymal transition (EMT) and invasive potential.

To confirm that this signature reflects chemotherapy-associated transcriptional selection rather than intrinsic TNBC subtype biology, we compared cancer epithelial cells from treatment-naïve TNBC versus non-TNBC samples from the same atlas—in which *S100A6* and *FABP5* were not upregulated in naïve TNBC—confirming that the key signature genes are not pre-existing intrinsic TNBC subtype markers in treatment-naïve tumors (Supplementary Figure S1G and Table S2). In contrast, comparison of chemo-treated versus naïve TNBC epithelial cells demonstrated that *S100A6* and *FABP5* were among the top 10 most significantly upregulated genes in the post-chemotherapy residuum (adjusted *p* < 0.001), and EMT-associated transcription factors *HMGA2*, *SNAI2*, and *ZEB1* were all significantly elevated in the Chemo-treated residuum—confirming that the FA-EMT signature is specifically driven by chemotherapy selection pressure in TNBC (Supplementary Figure S1H and Table S3).

To resolve the functional heterogeneity within the malignant epithelial compartment and identify the specific cancer epithelial subset responsible for TNBC chemotherapy resistance, we performed high-resolution unsupervised sub-clustering, resolving four transcriptionally distinct populations (Figure 1J; Supplementary Figure S1I–K): a Fatty Acid–EMT co-expressing subset (FA-EMT), a Fatty Acid–Luminal hybrid subset (FA-Luminal), and two progressively differentiated luminal subtypes (Luminal_2 and Luminal_1), each defined by the aggregate expression of canonical program-specific markers (Supplementary Figure S1I–K). The FA-EMT subset was characterized by the simultaneous activation of EMT plasticity markers—including *ZEB1*, *HMGA2*, and *SNAI2*—and fatty acid metabolic genes—including *FABP5*, *FASN*, *ACACA*, and *CD36*—representing a transcriptionally hybrid state in which mesenchymal reprogramming and lipid metabolic adaptation are co-activated within the same malignant epithelial population. Critically, compositional analysis across chemotherapy groups (Figure 1K, Supplementary Figure S1J) revealed a selective and marked expansion of the FA-EMT and FA-Luminal populations specifically in the TNBC post-treatment residuum—consistent with the differential expression and gene ontology findings above—while the Chemo_non_TNBC cohort was predominantly composed of Luminal_1 and Luminal_2 subtypes. Together, these findings establish that the malignant epithelial residuum persisting after chemotherapy in TNBC is defined by the FA-EMT subset as the principal resistance-associated population, co-activating EMT-driven phenotypic plasticity and lipid metabolic reprogramming to provide the structural flexibility and energetic substrate required to evade and survive cytotoxic therapy.

### 2.2. Fatty Acid–EMT Co-Expression Is Significantly Associated with Chemotherapy Resistance and Predicts the Worst Survival Outcome in TNBC: Independent Bulk Transcriptomic Validation

The single-cell analysis identified selective enrichment of the FA-EMT epithelial subset—co-expressing fatty acid metabolic and EMT plasticity programs—in the chemotherapy-treated TNBC residuum. To determine whether these findings generalize beyond the single-cell cohort, we performed independent bulk transcriptomic validation across 277 chemotherapy-treated TNBC patients drawn from two external cohorts: GSE25066 (n = 170; 57 sensitive, 113 resistant) [14] and GSE58812 (n = 107; 31 sensitive, 76 resistant) [15]. S = 88, r = 189.

**Figure 2:**
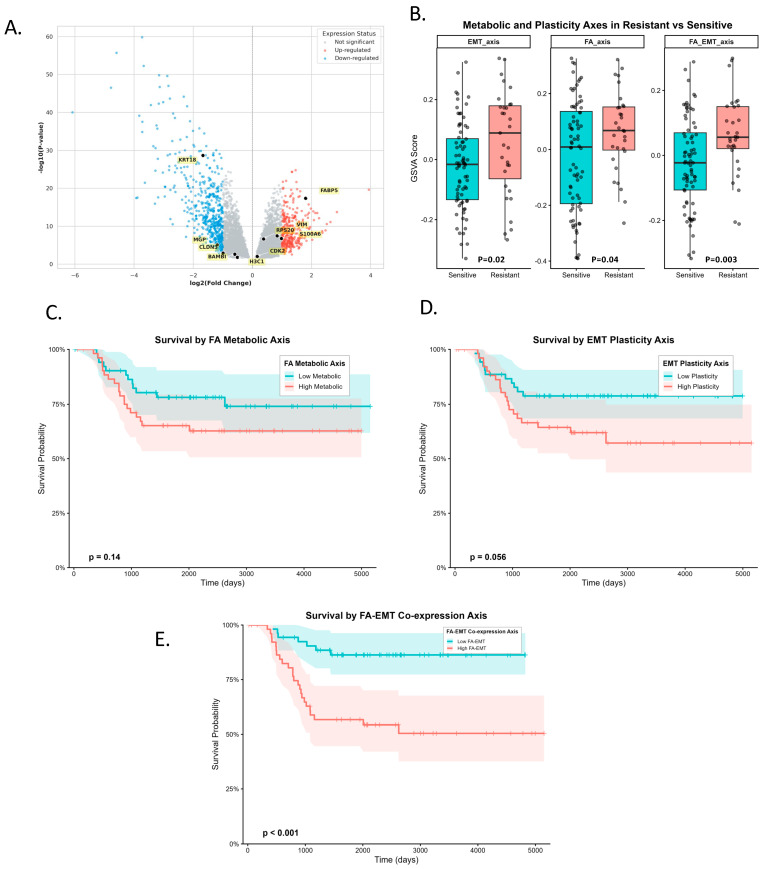
FA-EMT co-expression is significantly associated with chemotherapy resistance and poor survival outcomes in TNBC. Analyses were performed in independent bulk transcriptomic cohorts GSE25066 (n = 170; 57 sensitive, 113 resistant) and GSE58812 (n = 107; 76 sensitive, 31 resistant). (**A**) Volcano plot of differential gene expression between chemotherapy-resistant and chemotherapy-sensitive TNBC tumors in the GSE25066 cohort, plotted by log_2_(fold change) on the *x*-axis and −log_10_(*p*-value) on the *y*-axis. Red: upregulated genes in resistant tumors; blue: downregulated genes; gray: non-significant genes; black dots: highlighted candidate biomarker genes. Dashed vertical lines indicate the fold-change threshold; dashed horizontal line indicates the significance threshold. (**B**) Boxplots of Gene Set Variation Analysis (GSVA)-derived EMT Plasticity axis (left), FA Metabolic axis (middle), and FA-EMT co-expression axis (right) scores in the GSE58812 cohort, stratified by chemotherapy response (Sensitive, teal; Resistant, pink). *p*-values from Wilcoxon rank-sum test: *p* = 0.02, *p* = 0.04, and *p* < 0.001, respectively. (**C**) Kaplan–Meier overall survival curves stratified by FA Metabolic axis score (High_Metabolic, pink; Low_Metabolic, teal; median split). Log-rank *p*-value shown. (**D**) Kaplan–Meier overall survival curves stratified by EMT Plasticity axis score (High_Plasticity, pink; Low_Plasticity, teal; median split). Log-rank *p*-value shown. (**E**) Kaplan–Meier overall survival curves stratified by FA-EMT co-expression axis score (High_FA-EMT, pink; Low_FA-EMT, teal; median split). Log-rank *p*-value shown.

Differential expression analysis of the GSE25066 cohort comparing chemotherapy-resistant versus chemotherapy-sensitive TNBC patients’ tumors (Figure 2A and Supplementary Table S7) revealed a transcriptional profile consistent with the single-cell epithelial findings. Among the highlighted candidate biomarkers, *FABP5* and *S100A6* were upregulated in resistant tumors, corroborating the activation of lipid metabolic and stress-response programs identified at single-cell resolution. Conversely, *KRT18* and *MGP* were downregulated in the resistant group, indicating a loss of epithelial differentiation markers and a transcriptional shift consistent with acquisition of a phenotypically plastic, mesenchymal-like state. Additional resistance-associated genes, including *RPS20*, *S100A6*, *CDK2*, and *CLDN4B*, were also identified among the upregulated transcripts, further supporting broad activation of proliferative and plasticity-associated programs in chemorefractory TNBC.

To directly quantify the activity of the FA-EMT co-expression program and its two constituent axes—fatty acid metabolic reprogramming and EMT plasticity—in independent patient data, Gene Set Variation Analysis (GSVA) scoring was applied to the GSE58812 cohort using three gene signatures: the EMT Plasticity axis, the FA Metabolic axis, and a combined FA-EMT co-expression axis derived from the single-cell FA-EMT subset. Resistant tumors exhibited significantly higher scores across all three axes relative to sensitive tumors (EMT Plasticity axis: *p* = 0.02; FA Metabolic axis: *p* = 0.04; FA-EMT co-expression axis: *p* = 0.003; Figure 2B), confirming that the transcriptional programs defining the FA-EMT subset are reproducible molecular hallmarks of TNBC treatment failure in independent patient cohorts. Critically, the FA-EMT co-expression axis yielded the most significant separation between resistant and sensitive tumors (*p* = 0.003), demonstrating that the co-activation of both programs together is a stronger discriminator of chemotherapy resistance than either program individually—consistent with the hypothesis that FA-EMT represents a functionally integrated rather than a coincidental co-occurrence of two independent resistance mechanisms.

The clinical prognostic impact of each program was assessed by Kaplan–Meier survival analysis stratified by median GSVA score. Patients with high FA Metabolic axis activity showed a trend toward reduced overall survival compared with the low-metabolic group, although this association did not reach statistical significance (*p* = 0.14; Figure 2C), suggesting that metabolic reprogramming alone is insufficient as an independent prognostic determinant. High EMT Plasticity axis scores were associated with a more pronounced reduction in overall survival (*p* = 0.056; Figure 2D), indicating that epithelial–mesenchymal plasticity is the stronger individual prognostic driver of the two programs. Strikingly, however, stratification by the FA-EMT co-expression axis score yielded the most significant and clinically impactful survival separation of all three analyses: patients with high FA-EMT co-expression scores exhibited dramatically reduced overall survival compared with the low FA-EMT group (*p* < 0.001; Figure 2E), with the survival curves diverging sharply within the first 500 days and maintaining a sustained separation throughout the follow-up period. These findings establish a clear functional hierarchy: while fatty acid metabolic reprogramming and EMT plasticity each contribute independently to adverse prognosis, their co-expression within the FA-EMT subset amplifies their individual effects to produce the most clinically significant prognostic signal—underscoring the biological importance of the co-expressing subset as a unified driver of both treatment resistance and poor survival in TNBC.

Notably, stratified survival analysis separating patients by chemotherapy response and FA-EMT score level revealed that survival curves were similar between high- and low-FA-EMT subgroups within each response category, indicating that chemotherapy response itself is the dominant prognostic determinant in this cohort (Supplementary Figure S2A–C). However, a subset of chemotherapy-sensitive (pCR) patients carried high FA-EMT co-expression scores, suggesting that FA-EMT elevation is not exclusive to clinically resistant tumors and may identify a latent resistance-prone transcriptional state even among patients who initially achieve pathological complete response. These patients may warrant closer surveillance and could potentially benefit from FA-EMT-targeted intervention to prevent disease relapse, a hypothesis that warrants prospective investigation.

Collectively, these bulk transcriptomic validation analyses corroborate and extend the single-cell findings, establishing that chemotherapy-resistant TNBC is defined by the coordinated co-activation of fatty acid metabolic reprogramming and EMT plasticity—programs embodied at the cellular level by the FA-EMT epithelial subset identified in Figure 1—and that it is specifically the co-expression of these programs, rather than either alone, that most powerfully predicts treatment failure and unfavorable clinical prognosis.

### 2.3. The FA-EMT Epithelial Subset Co-Exists with Coordinated Immunosuppressive Remodeling of the Stromal, T-Cell, and Myeloid Tumor Microenvironment Compartments in Chemotherapy-Resistant TNBC

Having established that the malignant epithelial compartment of chemotherapy-resistant TNBC is dominated by the FA-EMT co-expressing subset, we next interrogated the non-malignant TME compartments—fibroblasts, T cells, and myeloid cells—to determine how the resistant microenvironment is coordinately remodeled at the stromal and immune levels. Each compartment was analyzed by sub-clustering, differential expression, and compositional profiling, comparing Chemo_TNBC and Chemo_non_TNBC cohorts (Supplementary Tables S4–S6, Figures S2D–G and S3A–H).

**Figure 3:**
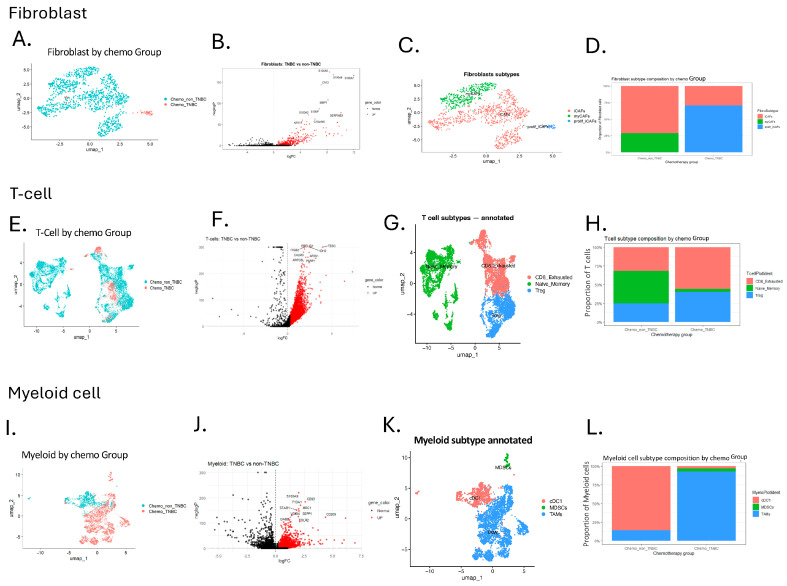
Remodeling of fibroblast, T-cell, and myeloid compartments in the chemotherapy-resistant TNBC tumor microenvironment. Each compartment was analyzed using UMAP, differential expression, sub-cluster annotation, and compositional profiling, comparing the Chemo_TNBC (resistant, R) and Chemo_non_TNBC (sensitive, S) cohorts. (**A**,**E**,**I**) UMAPs of fibroblast, T-cell, and myeloid cells, respectively, colored by chemotherapy group (Chemo_non_TNBC, teal; Chemo_TNBC, red). (**B**,**F**,**J**) Volcano plots of differential gene expression in fibroblasts, T cells, and myeloid cells, respectively, between Chemo_TNBC and Chemo_non_TNBC (Wilcoxon rank-sum test, Benjamini–Hochberg FDR correction). Red: upregulated in Chemo_TNBC; black: downregulated. Dashed lines indicate fold-change and significance thresholds. (**C**) Annotated sub-cluster UMAP of the fibroblast compartment identifying three subtypes: iCAFs (red), myCAFs (green), and prolif_iCAFs (blue). (**G**) Annotated sub-cluster UMAP of the T-cell compartment identifying three subtypes: CD8_Exhausted (red), Naive_Memory (green), and Treg (blue). (**K**) Annotated sub-cluster UMAP of the myeloid compartment identifying three subtypes: TAMs (red), cDC1 (teal), and MDSCs (blue). (**D**,**H**,**L**) Stacked bar plots of fibroblast, T-cell, and myeloid subtype proportions across chemotherapy groups, respectively.

Analysis of the cancer-associated fibroblast (CAF) compartment revealed a clear transcriptional divergence between the two groups (Figure 3A). Differential expression analysis (Figure 3B; Supplementary Table S4) identified *S100A8*, *S100A9*, *S100A7*, *SPRR1B*, *MMP1*, and *CLDN4* as among the most significantly upregulated genes in Chemo_TNBC fibroblasts. The co-upregulation of *S100A8* and *S100A9*—damage-associated molecular pattern molecules that activate the NF-κB inflammatory axis—together with MMP1 identifies a pro-inflammatory, matrix-remodeling program in TNBC-associated fibroblasts. High-resolution sub-clustering resolved the fibroblast compartment into three functionally distinct subtypes (Figure 3C; Supplementary Figure S2F,G): inflammatory CAFs (iCAFs), myofibroblastic CAFs (myCAFs), and proliferative inflammatory CAFs (prolif_iCAFs), each confirmed by canonical marker expression (Supplementary Figure S2G). Compositional analysis (Figure 3D) revealed a striking remodeling of the stromal landscape in Chemo_TNBC: the myCAF population—defined by contractile and desmoplastic markers including *ACTA2*, *TAGLN*, and *COL1A1*—was markedly depleted, while iCAF and prolif_iCAF subsets were disproportionately expanded. In contrast, the Chemo_non_TNBC cohort maintained a balanced stromal composition with a substantial myCAF fraction. This selective loss of myCAFs and dominance of pro-inflammatory iCAF states in resistant TNBC is consistent with a stromal niche that favors cytokine-mediated immune modulation—through iCAF secretion of *IL6*, *CXCL12*, and *CCL2*—over structural barrier formation, thereby promoting myeloid recruitment and T-cell exclusion.

Parallel analysis of the T-cell compartment demonstrated a pronounced immune transcriptional shift in Chemo_TNBC (Figure 3E). Differential expression analysis (Figure 3F; Supplementary Table S5) revealed broad upregulation of genes in the resistant group, with *TRGC2*, *TRDV2*, *AP2S1*, *TESC*, *CALM3*, and *PRELID1* among the most significantly elevated transcripts in Chemo_TNBC T cells, consistent with a dysfunctional, exhaustion-associated transcriptional state (Supplementary Figure S3A,B). Sub-clustering resolved the T-cell compartment into three populations (Figure 3G; Supplementary Figure S3C,D): CD8_Exhausted, Naive_Memory, and Regulatory T cells (Tregs), confirmed by canonical marker expression including *PRDM1*, *CALM3*, and *PRELID1* in the CD8_Exhausted subset, *CCR7*, *LEF1*, and *IL7R* in Naive_Memory cells, and *FOXP3*, *CTLA4*, and *TIGIT* in Tregs (Supplementary Figure S3D). Compositional analysis (Figure 3H) demonstrated that Chemo_TNBC tumors harbored a markedly higher proportion of CD8_Exhausted and Treg cells relative to Chemo_non_TNBC, in which the Naive_Memory T-cell fraction predominated. The expansion of CD8_Exhausted T cells in the resistant group reflects a dysfunctional, terminally exhausted state incapable of productive cytotoxic activity, while Treg enrichment establishes an active immunosuppressive axis that further neutralizes effective anti-tumor immunity within the TNBC microenvironment.

The myeloid compartment similarly exhibited subtype-specific transcriptional reprogramming in Chemo_TNBC (Figure 3I). Differential expression analysis (Figure 3J; Supplementary Table S6) identified *S100A9*, *FCAR*, *FOLR2*, *STAT1*, *NR1*, *SEPP1*, and *EDLR2* as significantly upregulated in resistant myeloid cells, consistent with activation of innate immune suppression and alternative macrophage polarization programs (Supplementary Figure S3E,F). High-resolution sub-clustering resolved the myeloid compartment into three subtypes (Figure 3K; Supplementary Figure S3G,H): tumor-associated macrophages (TAMs), conventional dendritic cells type 1 (cDC1), and myeloid-derived suppressor cells (MDSCs), each confirmed by canonical marker expression including *APOE*, *C1QA*, *TREM2*, and *FOLR2* in TAMs, *CLEC9A*, *XCR1*, and *BATF3* in cDC1, and *S100A8*, *S100A9*, and *FCN1* in MDSCs (Supplementary Figure S3H). Compositional analysis (Figure 3L) revealed that Chemo_non_TNBC tumors were dominated by cDC1—the myeloid subset most critical for cross-presentation of tumor antigens and priming of cytotoxic T-cell responses. In striking contrast, Chemo_TNBC tumors exhibited a near-complete depletion of cDC1 cells, with the myeloid compartment instead overwhelmingly dominated by TAMs and MDSCs. This cDC1-to-TAM/MDSC shift represents a fundamental impairment of innate immune surveillance: the loss of professional antigen-presenting cells disrupts priming of adaptive anti-tumor immunity, while TAM and MDSC expansion actively promote an immunosuppressive, pro-tumorigenic milieu.

Taken together, the remodeling of all three non-malignant TME compartments in Chemo_TNBC—the pro-inflammatory iCAF-dominant stroma, the CD8_Exhausted and Treg-enriched T-cell niche, and the cDC1-depleted, TAM/MDSC-enriched myeloid landscape—defines a broadly immunosuppressive microenvironment that acts in concert with the FA-EMT epithelial subset identified in Figure 1 and Figure 2 to establish and maintain chemotherapy resistance in TNBC. The convergence of these multi-compartment immunosuppressive signals with the FA-EMT epithelial program raises a central mechanistic question: does the FA-EMT subset actively orchestrate this TME remodeling through direct intercellular signaling, or does it merely co-exist within an independently established immunosuppressive niche? We address this question directly in the following section.

### 2.4. The FA-EMT Epithelial Subset Functions as the Dominant Intercellular Signaling Hub, Reprogramming the Immune Microenvironment via MIF–CD74–CXCR4 and MDK–NCL Ligand–Receptor Axes to Drive Chemotherapy Resistance in TNBC

Having identified coordinated remodeling of the epithelial, stromal, and immune compartments in chemotherapy-resistant TNBC, we next sought to determine whether the FA-EMT subset actively orchestrates this immunosuppressive TME remodeling through direct intercellular signaling. To this end, we performed high-resolution cell–cell communication analysis using the CellChat framework, mapping ligand–receptor (L–R) interaction profiles across all cell types and comparing signaling information flow between Chemo_TNBC (T) and Chemo_non_TNBC (N) groups, with a specific focus on interactions originating from the cancer epithelial sub-clusters (Supplementary Tables S8–S11).

**Figure 4:**
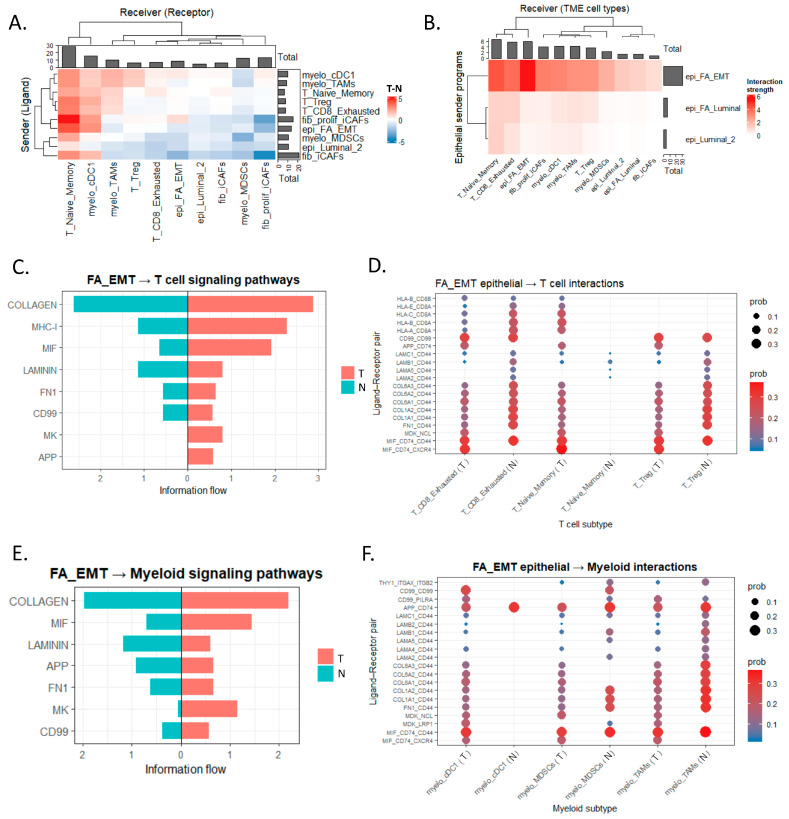
The FA-EMT epithelial subset reprograms the tumor microenvironment through ligand–receptor signaling to promote chemotherapy resistance in TNBC. Cell–cell communication analysis was performed using the CellChat framework, comparing the Chemo_TNBC resistant (T) and Chemo_non_TNBC sensitive (T) groups. (**A**) Heatmap of differential cell–cell interaction strength (T–N) across all cell types. Red: interactions enriched in resistant (T) tumors; blue: interactions enriched in sensitive (N) tumors. Bar plots indicate total incoming (top) and outgoing (right) interaction strength per cell type. (**B**) Focused heatmap of outgoing interaction strength restricted to epithelial sub-clusters (epi_FA_EMT, epi_FA_Luminal, epi_Luminal_2) as senders across all TME receiver cell types, colored by interaction strength (red, high; white, low). (**C**) Diverging bar plot of the top signaling pathway information flow from FA-EMT epithelial cells to T-cell subsets, comparing resistant (T, pink) and sensitive (N, teal) tumors. Pathway names are listed on the *y*-axis; bar length reflects information flow magnitude. (**D**) Bubble plot of FA-EMT epithelial → T-cell ligand–receptor interactions across T-cell subtypes and response groups (R, resistant; S, sensitive). Dot size indicates communication probability; dot color reflects probability magnitude (red, high; blue, low). (**E**) Diverging bar plot of the top signaling pathway information flow from FA-EMT epithelial cells to myeloid subsets, comparing resistant (T, pink) and sensitive (N, teal) tumors. (**F**) Bubble plot of FA-EMT epithelial → myeloid ligand–receptor interactions across myeloid subtypes and response groups (T, Chemo_TNBC; N, Chemo_nonTNBC). Dot size indicates communication probability; dot color reflects probability magnitude (red, high; blue, low).

Differential interaction strength analysis across all cell types (Figure 4A; T–N) revealed a pronounced shift in signaling architecture between resistant and sensitive tumors. In Chemo_TNBC (T) tumors, the strongest enriched interactions (indicated in red) converged on T_Naive_Memory, T_Treg, T_CD8_Exhausted, and myeloid populations—specifically myelo_cDC1 and myelo_TAMs—as primary receivers. Strikingly, epi_FA_EMT emerged as the dominant sender, generating the highest outgoing interaction strength of any cell type in the resistant TME (Figure 4B). In Chemo_non TNBC (N) tumors, communication was more broadly distributed and less immune-targeted (blue interactions), reflecting a fundamentally different TME signaling landscape in which the FA-EMT signaling dominance was absent.

To delineate the specific contribution of each epithelial subpopulation to this resistance-associated network, we restricted the sender compartment to the three epithelial sub-clusters—epi_FA_EMT, epi_FA_Luminal, and epi_Luminal_2—and re-examined outgoing interaction strengths across all TME receiver populations in the Chemo_TNBC group (Figure 4B). This analysis unequivocally identified epi_FA_EMT cells as the primary signaling hub within the epithelial compartment, exhibiting the highest outgoing interaction strength toward every TME cell type examined—including T cells, myeloid populations, fibroblasts, and other epithelial clusters. The epi_FA_Luminal and epi_Luminal_2 subtypes contributed substantially lower outgoing signal, further establishing the FA-EMT program as the principal driver of epithelial–TME crosstalk in chemotherapy-resistant TNBC.

We next characterized the specific signaling pathways mediating communication between *epi_FA_EMT* cells and the T-cell compartment. We identified a total of 27 FA-EMT → T cell signaling pathways (18 in the Chemo-TNBC [T] group and 9 in the Chemo-non-TNBC [N] group; Supplementary Table S6). The top pathway with information flow > 0.5 in either group is shown as a diverging bar plot (Figure 4C), enabling direct comparison of T-enriched, N-enriched, and shared signaling programs across the full FA-EMT → T cell pathway landscape. Structural and ECM pathways—including COLLAGEN (T: 2.89; N: 2.62), LAMININ, and FN1—showed the highest absolute information flow in both groups, reflecting shared epithelial–immune crosstalk present regardless of molecular subtype. Comparison of signaling information flow revealed that the most selectively Chemo-TNBC (T)-enriched immunomodulatory pathways were MIF (T: 1.92 vs. N: 0.65; 2.9-fold enriched), MK (T: 0.80 vs. N: absent), and APP (T: 0.59 vs. N: absent)—with MIF and MK enriched exclusively or predominantly in the T group against this shared structural background (Figure 4C and Supplementary Table S8)—indicating that *epi_FA_EMT* epithelial cells deploy a broad and intense immunomodulatory and extracellular matrix–remodeling signaling repertoire toward T cells that is specifically activated in the TNBC post-chemotherapy context. Detailed L–R interaction analysis at the molecular level (Figure 4D; Supplementary Table S9) identified three Chemo-TNBC (T)-specific receptor axes—each corresponding to a T-enriched pathway—that were exclusively detected in chemo-treated TNBC (T) with high communication probability: *MIF–CD74–CXCR4*, *MDK–NCL*, and *APP–CD74*. These interactions targeted T_CD8_Exhausted, T_Naive_Memory, and T_Treg subsets, and were markedly absent in the chemo-treated non-TNBC (N) group. These findings demonstrate that *epi_FA_EMT* cells deploy MIF and MDK as paracrine ligands that engage CD74, CXCR4, and NCL on T cells to induce a transcriptional exhaustion and immunosuppressive program within the adaptive immune compartment—providing a direct mechanistic explanation for the CD8_Exhausted and Treg phenotypes observed in (Figure 3G,H).

A parallel analysis of *epi_FA_EMT*–myeloid communication revealed a consistent and even more pronounced pattern of Chemo-TNBC (T)-enriched signaling (Figure 4E). Similarly, we identified a total of 40 FA-EMT → Myeloid signaling pathways (25 in T and 15 in N; Supplementary Table S10). The top pathways with information flow > 0.5 in either group are shown as a diverging bar plot (Figure 4E). Within this landscape, MIF (T: 1.45 vs. N: 0.69; 2.1-fold enriched) and MK (T: 1.16 vs. N: 0.05; 23-fold enriched) pathways showed markedly higher information flow in chemo-treated TNBC (T), mirroring the T-cell signaling landscape and confirming that the *FA-EMT* epithelial program simultaneously coordinates immunosuppressive crosstalk across both adaptive and innate immune compartments. The 23-fold enrichment of MK signaling in the T-group myeloid compartment is particularly striking and reinforces *MDK–NCL* as the most selectively TNBC-associated axis in innate immune suppression. Molecular-level L–R interaction analysis (Figure 4F; Supplementary Table S11) identified *MIF–CD74–CXCR4* and *MDK–NCL* as the most prominently and consistently T-enriched interactions across all myeloid subtypes—myelo_cDC1, myelo_MDSCs, and myelo_TAMs—while these interactions were absent or negligible in the chemo-treated non-TNBC (N) group. Notably, the selective targeting of cDC1—the myeloid population essential for tumor antigen cross-presentation and priming of cytotoxic CD8 T-cell responses—by MIF and MDK ligands from *epi_FA_EMT* cells provides a direct mechanistic basis for the cDC1 depletion and functional impairment of innate immune surveillance observed in the chemo-treated TNBC (T) TME (Figure 3K,L), directly linking the FA-EMT signaling program to the innate immune collapse that characterizes chemotherapy-resistant TNBC.

Collectively, these findings establish that *epi_FA_EMT* cells function as a master intercellular signaling hub in chemotherapy-resistant TNBC, orchestrating a dual immunosuppressive program through the coordinated deployment of MIF and MK pathway ligands. These enrichment ratios establish *MDK–NCL* and *MIF–CD74–CXCR4* as the most specifically Chemo-TNBC (T)-associated signaling axes within the broader FA-EMT signaling repertoire—against a background of shared structural pathways present in both groups—justifying their nomination as the principal mechanistically grounded therapeutic targets. By simultaneously targeting the adaptive immune compartment—inducing T-cell exhaustion and Treg activation via *MIF–CD74–CXCR4* and *APP–CD74*—and the innate immune compartment—impairing cDC1-mediated antigen presentation and expanding MDSCs and TAMs via *MDK–NCL* and *MIF–CD74–CXCR4*—the *FA-EMT* epithelial program constructs a comprehensively immunosuppressive niche that protects residual tumor cells from immune-mediated elimination and sustains chemotherapy resistance in TNBC. These ligand–receptor axes therefore represent mechanistically grounded therapeutic targets with the potential to sensitize chemotherapy-resistant TNBC to cytotoxic treatment.

### 2.5. Spatial Multiplexed Protein Imaging Validates the Metabolic-EMT Subset and Its Immunosuppressive Co-Localization in Chemotherapy-Resistant TNBC Model

Having established through single-cell transcriptomics and CellChat ligand–receptor analysis that the FA-EMT epithelial subset functions as a master immunosuppressive signaling hub in chemotherapy-resistant TNBC, a critical question remained: does this subset physically exist and spatially co-localize with the immunosuppressive cell populations it was predicted to recruit, within the architecture of resistant tumor tissue? To address this directly, we leveraged published multiplexed protein imaging data (CyCIF) from a previously characterized syngeneic TNBC *mouse* model [16]. In this model, two tumor lines were established with intrinsically distinct stromal phenotypes—stroma-poor (SP; n = 26 cores) and stroma-rich (SR; n = 43 cores)—that were not derived by paclitaxel treatment but were subsequently shown to exhibit a consistent differential response to paclitaxel: SP tumors are intrinsically paclitaxel-resistant while SR tumors remain paclitaxel-sensitive [16]. These two lines, therefore, represent established paclitaxel-resistant and paclitaxel-sensitive phenotypes, enabling spatial validation of our computational findings at single-cell protein resolution within intact tumor tissue.

**Figure 5:**
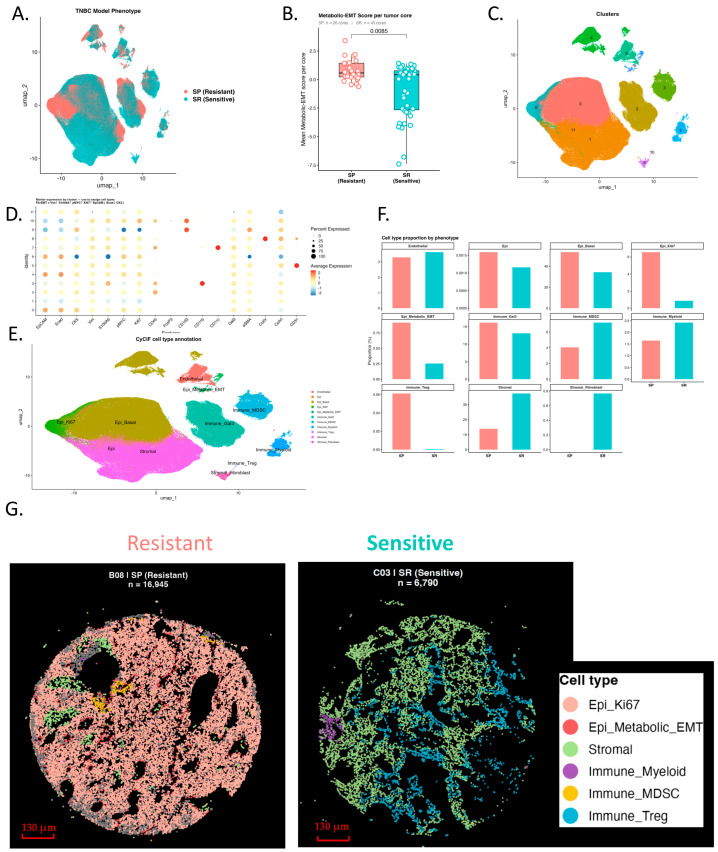
Spatial validation of the Metabolic-EMT subset and immunosuppressive co-localization in a previously published TNBC mouse model using multiplexed protein imaging (CyCIF) analysis. CyCIF analysis was performed on the tissue microarray of a publicly available TNBC mouse [16], comprising SP (stroma-poor, paclitaxel-resistant phenotype; n = 26 cores) and SR (stroma-rich, paclitaxel-sensitive phenotype; n = 43 cores) tumors. (**A**) UMAP of all CyCIF-profiled cells colored by tumor phenotype (SP-resistant, coral; SR-sensitive, teal). (**B**) Boxplot of mean Metabolic-EMT score per tumor core (SP: n = 26 cores; SR: n = 43 cores). Each dot represents one tumor core. Metabolic-EMT score was computed as the sum of arcsinh-normalized Vimentin, S100A6, pMYC, and Ki67 expression. *p*-value from the Wilcoxon rank-sum test. (**C**) Unsupervised clustering identitied 12 clusters (0–11), UMAP colored by cluter. (**D**) Dot plot of canonical marker expression across clusters used for manual cell type annotation. Dot size: percentage of expressing cells; color intensity: scaled mean expression. (**E**) Annotated UMAP colored by assigned cell type. (**F**) Bar plots of cell type proportions in SP versus SR tumors, showing selective enrichment of Epi_Metabolic_EMT, Epi_Ki67, and Immune_Treg in SP-resistant tumors, and depletion of Stromal compartments in resistant relative to sensitive tumors. (**G**) Representative spatial pseudo-images of SP-resistant (B08) and SR-sensitive (C03) tumor cores. Cells are colored by selected cell types to highlight spatial co-localization of the Epi_Metabolic_EMT subset (red) and proliferating Epi_Ki67 cells (salmon) with immunosuppressive populations—Immune_MDSC (gold) and Immune_Treg (teal)—selectively in resistant tumors. Background cells are shown in dark gray. Scale bar = 130 µm.

UMAP dimensionality reduction of 565,438 CyCIF-profiled cells demonstrated a clear separation between SP-resistant and SR-sensitive tumor cells, with SP cells concentrated within transcriptionally restricted clusters and SR cells broadly distributed—mirroring the separation observed in the scRNA-seq UMAP in Figure 1A and confirming phenotype-specific transcriptional restriction at the protein level (Figure 5A). To quantify the activity of the Metabolic-EMT program across tumors, we computed a composite Metabolic-EMT score per cell as the sum of arcsinh-normalized Vimentin, S100A6, pMYC, and Ki67 expression. These four markers were selected as the best available CyCIF proxies for the FA-EMT transcriptional signature identified in Figure 1: Vimentin directly marks mesenchymal identity and EMT, S100A6 is co-upregulated with *FABP5* in the FA-EMT subset and serves as a surrogate of lipid metabolic stress and calcium-dependent signaling, pMYC reflects MYC-driven transcriptional activation of fatty acid biosynthesis genes including *FASN* and *ACACA*, and Ki67 marks the proliferative arm of the FA-EMT co-expression program. Together, Vim+ S100A6+ pMYC+ Ki67+ co-expression captures both the mesenchymal and metabolic dimensions of the FA-EMT identity at the protein level. This Metabolic-EMT score was significantly elevated in SP-resistant tumor cores relative to SR-sensitive cores (*p* = 0.0085, Wilcoxon rank-sum test; Figure 5B), confirming that the Metabolic-EMT protein program is a reproducible and statistically significant feature of chemotherapy resistance at the tissue level.

Unsupervised clustering of all profiled cells identified 12 phenotypically distinct populations (Figure 5C), annotated into epithelial, immune, and stromal lineages based on canonical marker expression across clusters (Figure 5D,E). Among the epithelial clusters, a discrete population was identified by co-expression of Vimentin, S100A6, pMYC, and Ki67 with low EpCAM and E-cadherin—directly mirroring the protein-level signature of the FA-EMT subset identified transcriptomically in Figure 1, representing EMT identity (Vimentin, low EpCAM/E-cadherin), lipid metabolic stress (S100A6), MYC-driven fatty acid biosynthesis (pMYC), and proliferative activation (Ki67). This population is hereafter designated Epi_Metabolic_EMT, reflecting its protein-level definition as the spatial CyCIF counterpart of the transcriptomic FA-EMT subset.

Compositional analysis across all 69 tumor cores revealed that the Epi_Metabolic_EMT subset was selectively and markedly enriched in SP-resistant tumors relative to SR-sensitive tumors, while SR tumors were dominated by Stromal and Stromal_Fibroblast populations—consistent with the stromal-rich, immune-diverse architecture of chemotherapy-sensitive tumors (Figure 5F). This enrichment was accompanied by a coordinated shift in the immune compartment: SP-resistant tumors harbored elevated proportions of Immune_Treg and Immune_MDSC populations alongside Epi_Ki67 expansion, while SR-sensitive tumors showed higher stromal representation—directly mirroring the multi-compartment TME remodeling identified computationally in Figure 3 and Figure 4.

Most compellingly, spatial pseudo-image analysis of SP-resistant and SR-sensitive tumor cores revealed striking differences in the spatial organization of these populations within intact tissue (Figure 5G; Supplementary Figure S4). For Figure 5G, representative cores were selected to reflect the median Metabolic-EMT score within each group. In SP-resistant cores—Epi_Metabolic_EMT and Epi_Ki67 cells were abundantly present and spatially interdigitated with Immune_MDSC and Immune_Treg populations throughout the tumor parenchyma, establishing physical proximity between the Metabolic-EMT epithelial program and the immunosuppressive immune populations it was predicted to recruit through MDK–NCL and MIF–CD74–CXCR4 signaling. In striking contrast, SR-sensitive cores were dominated by Stromal cells with sparse Epi_Metabolic_EMT representation and minimal Immune_Treg presence, consistent with an immune-permissive, stromal-rich architecture incompatible with sustained chemotherapy resistance.

Collectively, these spatial protein imaging findings provide direct tissue-level validation of the central mechanistic model proposed in this study: the FA-EMT/Metabolic-EMT epithelial subset is selectively enriched in chemotherapy-resistant TNBC tumors, physically co-localizes with immunosuppressive MDSC and Treg populations in resistant tumor tissue, and its abundance is significantly associated with the resistant phenotype across independent tumor cores. These data bridge the gap between computational ligand–receptor predictions and spatial tissue reality, establishing that the immunosuppressive niche constructed by FA-EMT cells through MDK–NCL and MIF–CD74–CXCR4 signaling is not merely a transcriptional prediction but a physically organized, spatially confirmed resistance architecture in TNBC.

## 3. Discussion

This study employed an integrative single-cell, bulk transcriptomic, and spatial proteomic framework to interrogate the cellular and molecular basis of chemotherapy resistance in TNBC. By leveraging a publicly available scRNA-seq atlas of NAC-treated breast tumors, two treatment-naïve validation analyses confirming that the FA-EMT signature is chemotherapy-driven rather than intrinsic to the TNBC subtype, independent bulk transcriptomic validation across 277 TNBC patients, and spatial CyCIF validation in a published paclitaxel-resistant TNBC mouse model, we identify a residual Fatty Acid–EMT co-expressing epithelial subset—FA-EMT—as a key cellular driver of chemotherapy resistance that actively reprograms the surrounding immune microenvironment through coordinated MDK–NCL and MIF–CD74–CXCR4 ligand–receptor signaling. Critically, we demonstrate that it is specifically the co-expression of fatty acid metabolic reprogramming and EMT plasticity within this discrete subset—rather than either program individually—that most powerfully predicts chemotherapy resistance and adverse clinical outcome. Spatial CyCIF validation further confirms that FA-EMT cells are not only transcriptionally predicted but physically present and spatially co-localized with immunosuppressive MDSC and Treg populations in resistant tumor tissue, converging on a mechanistic model in which the FA-EMT subset constructs a comprehensively immunosuppressive niche that shields residual tumor cells from cytotoxic elimination.

The identification of the FA-EMT subset as the dominant post-chemotherapy epithelial subpopulation in TNBC is consistent with, and substantially extends, a growing body of evidence that chemotherapy resistance is not a genetically predetermined trait uniformly distributed across all tumor cells, but rather an emergent property of specific transcriptional states that confer phenotypic fitness under therapeutic stress [6,7]. Prior bulk transcriptomic studies identified EMT-associated gene signatures as predictors of poor response to NAC in breast cancer [4,5], but were unable to resolve which specific cellular subpopulation harbored these programs or how they were organized within the broader tumor cellular hierarchy. Our single-cell analysis resolves this ambiguity by demonstrating that EMT plasticity and fatty acid metabolic reprogramming are co-expressed within the same epithelial subset—a finding that challenges both the classical “go or grow” dichotomy in which EMT and cell-cycle activation were considered mutually exclusive [17,18,19], and the prevailing assumption that metabolic and mesenchymal resistance programs operate independently. Importantly, our validation analyses demonstrate that this co-expression is not a baseline feature of TNBC but is specifically induced by chemotherapy exposure, consistent with a treatment-selected transcriptional state rather than a pre-existing subclonal population. The simultaneous activation of both programs in FA-EMT cells confers a dual advantage: the fatty acid metabolic component provides a high-yield energetic substrate required to sustain survival under cytotoxic stress, while the mesenchymal identity confers apoptotic resistance, phenotypic plasticity, and immune evasion capacity. While the interplay between metabolic reprogramming and EMT plasticity has been increasingly recognized—with evidence suggesting that each program can drive the other in a mutually reinforcing manner across multiple cancer types, including breast, colorectal, and other solid tumors [20,21,22]—their simultaneous co-expression within a single, discrete, residual epithelial subset and the specific association of this co-expression with post-chemotherapy survival in TNBC has not previously been demonstrated.

The bulk transcriptomic validation data provide compelling evidence for the clinical primacy of the FA-EMT co-expression program. While the FA Metabolic axis alone showed only a trend toward reduced survival (*p* = 0.14) and the EMT Plasticity axis alone reached borderline significance (*p* = 0.056), the FA-EMT co-expression axis yielded dramatically superior prognostic discrimination (*p* < 0.001), with survival curves diverging sharply within the first 500 days and maintaining sustained separation throughout follow-up. This synergistic prognostic impact of co-expression over individual programs establishes a functional hierarchy in which it is the integration—rather than simple summation—of fatty acid metabolic reprogramming and EMT plasticity that constitutes the primary determinant of clinical outcome in chemotherapy-resistant TNBC. This hierarchical relationship has not previously been demonstrated in TNBC and has important implications for therapeutic prioritization: strategies that simultaneously target both the metabolic and mesenchymal components of the FA-EMT program are likely to offer substantially greater clinical benefit than targeting either axis alone.

The spatial CyCIF validation performed in this study provides an important bridge between the transcriptional and clinical findings described above and direct tissue-level evidence. Using published multiplexed protein imaging data from a paclitaxel-resistant TNBC mouse model [16], we demonstrate that a Metabolic-EMT protein signature—defined by co-expression of Vimentin, S100A6, pMYC, and Ki67 with loss of EpCAM and E-cadherin, reflecting mesenchymal transition and metabolic activation at the protein level—was significantly elevated in resistant SP tumor cores relative to sensitive SR cores (*p* = 0.0085). Critically, spatial pseudo-image analysis revealed that Epi_Metabolic_EMT cells are not randomly distributed within resistant tumors but are physically interdigitated with Immune_MDSC and Immune_Treg populations throughout the tumor parenchyma—directly confirming the spatial proximity between the FA-EMT epithelial program and the immunosuppressive populations it was predicted to recruit through MDK–NCL and MIF–CD74–CXCR4 signaling. In contrast, sensitive SR cores were dominated by stromal cells with sparse Metabolic-EMT representation and negligible Treg infiltration, consistent with an immune-permissive architecture. These spatial findings are particularly significant because they transform the mechanistic model from a computationally inferred signaling prediction to a spatially confirmed tissue reality—directly establishing that the immunosuppressive niche constructed by FA-EMT cells is physically organized within resistant TNBC tumor architecture.

The TME remodeling we observe in chemotherapy-resistant TNBC extends well beyond the malignant epithelial compartment. The near-complete depletion of myCAFs and the selective expansion of iCAFs in the resistant group are particularly striking findings. myCAFs are classically associated with desmoplastic barrier formation through dense collagen deposition, while iCAFs are defined by their secretion of pro-inflammatory cytokines, including IL-6, CXCL12, and CCL2 [23]. The loss of myCAFs and dominance of iCAFs in resistant TNBC suggests a fundamental remodeling of the stromal niche away from structural containment and toward paracrine immune modulation—a shift that would be expected to facilitate both tumor cell dissemination and immune exclusion through sustained myeloid recruitment and T-cell suppression. This CAF compositional shift aligns with emerging evidence from spatial transcriptomic studies demonstrating that iCAF-dominant stromal niches are associated with immune-excluded tumor phenotypes and worse clinical outcomes in pancreatic and breast cancer [24,25,26], and our data extend this concept specifically to the post-chemotherapy TNBC context.

Within the immune compartments, our findings demonstrate a coordinated, multi-layered immunosuppressive architecture. The expansion of CD8_Exhausted T cells and Tregs in resistant tumors, alongside the near-complete depletion of cDC1 and expansion of TAMs and MDSCs in the myeloid compartment, is consistent with the establishment of a dysfunctional immune state in which both innate priming and adaptive cytotoxicity are simultaneously impaired. cDC1 depletion is particularly mechanistically significant: cDC1 constitutes the principal myeloid population responsible for cross-presenting tumor-derived antigens to CD8 T cells, and its loss has been directly linked to failed anti-tumor immunity and immunotherapy resistance in multiple cancer types [27]. Our data establish that cDC1 depletion in resistant TNBC is not merely a passive consequence of the immunosuppressive environment but is instead actively orchestrated by epi_FA_EMT cells through MDK–NCL and MIF–CD74–CXCR4 signaling—a mechanistic finding with direct therapeutic implications that directly links the FA-EMT epithelial program to innate immune collapse.

The ligand–receptor interaction analysis using CellChat provides the critical mechanistic link between the FA-EMT epithelial program and the immunosuppressive TME remodeling observed across all compartments. The identification of epi_FA_EMT cells as the dominant intercellular signaling hub—with the highest outgoing interaction strength of any epithelial sub-cluster toward every TME cell type examined—establishes that these cells are not passive bystanders in the resistance process but active architects of the microenvironment. The two key signaling axes we identify, MDK–NCL and MIF–CD74–CXCR4, are both biologically plausible and clinically actionable. MIF is a pleiotropic cytokine that signals through the CD74–CXCR4 receptor complex to promote immune evasion, tumor survival, and myeloid recruitment across multiple cancer types [28,29]. MDK is a heparin-binding growth factor that engages nucleolin (NCL) on the cell surface to promote cancer cell survival, inhibit apoptosis, and suppress antigen-presenting cell function [30]. While both pathways have been individually implicated in cancer biology, our study is the first to demonstrate their coordinated deployment by a specific FA-EMT co-expressing epithelial subset to simultaneously suppress both adaptive immunity—via CD8 T-cell exhaustion and Treg activation through MIF–CD74–CXCR4, MDK–NCL, and APP–CD74—and innate immune surveillance—via cDC1 impairment and MDSC/TAM expansion through MDK–NCL, and MIF–CD74–CXCR4—in the post-chemotherapy TNBC microenvironment. This dual-axis immunosuppressive mechanism provides a comprehensive explanation for why the immune system fails to eliminate residual tumor cells following chemotherapy in TNBC.

From a translational perspective, these findings nominate MDK–NCL and MIF–CD74–CXCR4 as mechanistically grounded therapeutic targets in chemotherapy-resistant TNBC. Several pharmacological strategies for targeting these axes are already in various stages of clinical and preclinical development. Small-molecule MIF inhibitors, including ISO-1 and 4-IPP, have demonstrated anti-tumor activity in preclinical models of breast and other cancers [29,30], and anti-CD74 antibody-drug conjugates are under clinical investigation. Similarly, iSONEP and other NCL-targeting agents have shown preclinical efficacy in suppressing tumor growth and immune evasion [28,30,31]. The mechanistic rationale our data provide—that disrupting these axes would simultaneously dismantle the protective immune niche constructed by epi_FA_EMT cells and restore both innate and adaptive anti-tumor immunity—suggests that combining MIF or MDK pathway inhibitors with conventional chemotherapy could sensitize resistant TNBC tumors to cytotoxic treatment by restoring immune surveillance prior to or concurrently with chemotherapy administration. Given that the FA-EMT co-expression program most powerfully predicts resistance and survival, FA-EMT scoring may additionally serve as a clinically actionable biomarker to identify patients most likely to benefit from such combination strategies. These hypotheses warrant prospective validation in preclinical TNBC models and ultimately in clinical trials.

Several important future directions emerge from this study. Prospective collection of larger, clinically annotated single-cell cohorts from chemotherapy-treated TNBC patients would further extend the cellular resolution achieved here. Functional experimental confirmation of the MDK–NCL and MIF–CD74–CXCR4 axes through ligand blocking, co-culture experiments, and in vivo TNBC models will be essential to establish causality and therapeutic potential. Finally, future spatial transcriptomic studies in human TNBC with matched pre- and post-treatment samples would confirm the clinical generalizability of the spatial co-localization findings. Together, these directions provide a clear roadmap for translating the mechanistic insights identified here into therapeutic applications in chemotherapy-resistant TNBC.

In conclusion, this study identifies the FA-EMT co-expressing epithelial subset as a chemotherapy-driven, immunosuppressive cellular program that predicts resistance and poor survival in TNBC more powerfully than either fatty acid or EMT programs alone. MDK–NCL and MIF–CD74–CXCR4 are established as the principal ligand–receptor axes through which FA-EMT cells actively construct an immunosuppressive niche, validated across single-cell, bulk transcriptomic, and spatial proteomic platforms. These findings nominate the FA-EMT subset and its signaling axes as mechanistically grounded therapeutic targets to overcome chemotherapy resistance in TNBC.

## 4. Materials and Methods

### 4.1. Data Sources and Study Design

This study employed a multi-platform transcriptomic and spatial proteomic framework integrating single-cell RNA sequencing (scRNA-seq), bulk RNA sequencing (RNA-seq), and multiplexed protein imaging (CyCIF) data to investigate the cellular and molecular mechanisms of chemotherapy resistance in triple-negative breast cancer (TNBC). All data were obtained from publicly available repositories; no new patient samples were collected, and no ethical approval was required for this study.

Single-cell transcriptomic data were obtained from a publicly available, high-resolution scRNA-seq atlas of primary human breast cancers (Gene Expression Omnibus [GEO] accession: GSE176078 [13]). From this atlas, samples were filtered to retain only patients who received neoadjuvant chemotherapy (NAC), yielding a primary study cohort of five patients stratified by molecular subtype: TNBC (n = 2; CID4513, CID4523), and non-TNBC (n = 3; CID3963, CID4066, CID4398), hereafter designated Chemo_TNBC, and Chemo_non_TNBC. These five samples represent all Chemo-treated cases available in the Wu et al. [13]; the remaining samples in GSE176078 were treatment-naïve and were not included in the primary scRNA-seq comparison.

To validate that the key FA-EMT signature reflects chemotherapy-associated transcriptional selection rather than intrinsic TNBC subtype biology, two additional comparative analyses were performed using treatment-naïve samples from the same atlas:

Validation analysis 1—treatment-naïve TNBC versus treatment-naïve non-TNBC epithelial cells (Supplementary Figure S1G and Table S2; naïve TNBC [patients: CID4465, CID44971, CID4495, CID44991, CID4515] vs. naïve non-TNBC [patients: CID3586, CID3838, CID3921, CID3941, CID3948, CID4040, CID4067, CID4290, CID4461, CID4463, CID4471, CID4517-1, CID4530, CID4535]).

Validation analysis 2—chemo-treated versus treatment-naïve TNBC epithelial cells (Supplementary Figure S1H and Table S3; Chemo TNBC [patients: CID4513, CID4523] vs. naïve TNBC [patients: CID4465, CID44971, CID4495, CID44991, CID4515]).

For independent bulk transcriptomic validation, two publicly available datasets were obtained from GEO: GSE25066 (n = 170 chemotherapy-treated TNBC patients; 57 sensitive [pathological complete response, pCR] and 113 resistant [residual disease, RD]) [14] and GSE58812 (n = 107 TNBC patients; 76 sensitive and 31 resistant) [15]. Combined, these cohorts comprised 277 chemotherapy-treated TNBC patients. Treatment response was classified as sensitive (pCR) or resistant (RD) based on pathological outcome annotations available within each dataset’s clinical metadata.

For spatial proteomic validation, published multiplexed protein imaging (CyCIF) data were obtained from a previously published TNBC mouse model [16]. The tissue microarray dataset RS-mTMA-5 comprised 69 tumor cores after quality filtering: SP (stroma-poor, paclitaxel-resistant; n = 26 cores) and SR (stroma-rich, paclitaxel-sensitive; n = 43 cores). Raw mean intensity data were obtained from the published Supplementary Materials and processed as described in Section 4.7.

### 4.2. Single-Cell RNA Sequencing Data Processing

Raw count matrices from the scRNA-seq atlas were processed using the Seurat package (v4.3.0) in R (v4.2.0) [16]. Quality control was applied per sample using the following thresholds: The top highly variable genes (HVGs) were identified using the FindVariableFeatures function with the variance-stabilizing transformation (VST) method. Data were scaled using ScaleData, and dimensionality reduction was performed by principal component analysis (PCA) on the HVG set. The first 30 principal components were used for downstream graph-based clustering and Uniform Manifold Approximation and Projection (UMAP) visualization.

### 4.3. Cell Type Annotation and Sub-Clustering

Major cell lineages—including Cancer Epithelial, T cells, Myeloid cells, Cancer-Associated Fibroblasts (CAFs), Normal Epithelial, B cells, Plasmablasts, Endothelial, and Perivascular-Like (PVL) cells—were previously defined by [13]. The four major lineages of interest (Cancer Epithelial, T cells, Myeloid cells, and CAFs) were then independently sub-clustered at higher resolution to identify functionally distinct subpopulations. Unsupervised graph-based clustering was performed using the FindNeighbors and FindClusters functions in Seurat. Cancer epithelial sub-clusters were annotated into four functional subtypes based on aggregate expression of canonical program-defining markers (Supplementary Figure S1D,E): Fatty Acid–EMT (FA-EMT), Fatty Acid–Luminal (FA-Luminal), Luminal_2, and Luminal_1. The FA-EMT subtype was defined by co-expression of EMT plasticity markers—including *VIM*, *ZEB1*, *HMGA2*, and *SNAI2*—and fatty acid metabolic markers—including *FABP5*, *FASN*, *ACACA*, and *CD36*. T-cell sub-clusters were annotated as CD8_Exhausted, Naive_Memory, and Regulatory T cells (Tregs); myeloid sub-clusters as tumor-associated macrophages (TAMs), conventional dendritic cells type 1 (cDC1), and myeloid-derived suppressor cells (MDSCs); and fibroblast sub-clusters as inflammatory CAFs (iCAFs), myofibroblastic CAFs (myCAFs), and proliferative iCAFs (prolif_iCAFs), each confirmed by canonical marker expression (Supplementary Figure S1H,I and Supplementary Figure S2D,H).

### 4.4. Differential Expression Analysis and Functional Enrichment

Differential gene expression (DGE) analysis between chemotherapy groups was performed for each of the four cell compartments independently using Seurat’s FindMarkers function with the Wilcoxon rank-sum test. Genes were considered significantly differentially expressed at an absolute log_2_ fold-change threshold > 0.5 and a Benjamini–Hochberg (BH) adjusted *p*-value < 0.05 (Supplementary Dataset). Volcano plot and dot plot visualization were performed using ggplot2 (v3.4.2), dplyr (v1.1.2), and ggrepel (v0.9.3) in R software (v4.2.3). Gene ontology (GO) biological process enrichment analysis of significantly upregulated genes was performed using the clusterProfiler package (v4.6.0) in R (v4.2.3) [32], with BH correction applied for multiple testing. Results were visualized with dotplot(), and cnetplot() functions from the enrichplot package (v1.3.4).

### 4.5. Bulk Transcriptomic Validation

#### 4.5.1. Gene Set Variation Analysis

To quantify the activity of three resistance-associated gene signatures in independent bulk transcriptomic data—the EMT Plasticity axis, the FA Metabolic axis, and the FA-EMT co-expression axis, derived from the FA-EMT, FA-Luminal, and combined single-cell sub-clusters, respectively—Gene Set Variation Analysis (GSVA) was applied to the GSE58812 cohort using the GSVA package (v1.46.0) in R (v4.2.3) [33]. GSVA computes per-sample enrichment scores for each gene set in a non-parametric, unsupervised manner, enabling the quantification of pathway activity across individual patients without requiring a predefined phenotype contrast. Enrichment scores were computed using the Gaussian kernel method with default parameters. All three axes were compared between chemotherapy-resistant (RD) and chemotherapy-sensitive (pCR) patients using the Wilcoxon rank-sum test.

#### 4.5.2. Statistical Comparison Between Response Groups

GSVA enrichment scores for the EMT Plasticity axis, the FA Metabolic axis, and the FA-EMT co-expression axis were compared between resistant (RD) and sensitive (pCR) patient groups using the Wilcoxon rank-sum test. Statistical significance was set at *p* < 0.05. Group comparisons were visualized as boxplots with individual data points overlaid.

#### 4.5.3. Differential Expression in Bulk Cohorts

Differential expression analysis between chemotherapy-resistant (RD) and chemotherapy-sensitive (pCR) TNBC patients in the GSE25066 cohort was performed using the limma-voom framework [34]. Gene expression data were log_2_-transformed and quantile-normalized prior to analysis. Differentially expressed genes were visualized as volcano plots, with significance thresholds set at log_2_FC > 0.5 and adjusted *p* < 0.05.

#### 4.5.4. Survival Analysis

Overall survival (OS) analysis was performed in the GSE58812 cohort. Patients were stratified into high- and low-GSVA-score groups for each axis using the median score as the split threshold. Kaplan–Meier survival curves were generated and compared using the log-rank test, implemented via the survival and survminer packages in R (v4.2.3). *p*-values < 0.05 were considered statistically significant.

### 4.6. Cell–Cell Communication Analysis

Intercellular ligand–receptor interaction analysis was performed using the CellChat framework (v1.6.1) in R (v4.2.3) [12]. CellChat was applied separately to the resistant (TNBC) and sensitive (non-TNBC) single-cell datasets. For each dataset, a CellChat object was constructed from the normalized count matrix, with cell type annotations as input. The CellChat database of curated ligand–receptor interaction pairs was used to compute communication probabilities across all cell type pairs using the default truncated mean method (trim = 0.1). Differential interaction strength between resistant and sensitive groups was computed by subtracting sensitive-group interaction scores from resistant-group scores (R–S), and visualized as heatmaps of sender–receiver communication strength. Top signaling pathway information flow was compared between groups, and the top specific L–R interactions were visualized as bubble plots with dot size indicating communication probability and color indicating probability magnitude.

### 4.7. Spatial Proteomic Validation—CyCIF Analysis

Spatial validation was performed using published CyCIF multiplexed protein imaging data from the tissue microarray [16]. Raw background-subtracted mean intensity data were loaded and cells assigned to SP (stroma-poor, paclitaxel-resistant) or SR (stroma-rich, paclitaxel-sensitive) phenotypes based on core annotations from the original publication, yielding 565,438 cells across 69 assigned cores (SP: n = 26 cores; SR: n = 43 cores). Marker intensities were arcsinh-transformed using a cofactor of 5, a standard normalization for CyCIF data. A Seurat object was constructed using the cycif assay, and data were scaled and dimensionality-reduced by PCA (10 components), UMAP, and graph-based clustering (FindNeighbors k = 8, FindClusters resolution = 0.15, SLM algorithm). Clusters were manually annotated based on canonical marker expression using a dot plot of 18 protein markers. A composite Metabolic-EMT score was computed per cell as the sum of arcsinh-normalized Vimentin, S100A6, pMYC, and Ki67 expression—selected as the best available CyCIF proxies for the FA-EMT transcriptional signature, representing EMT identity (Vimentin), lipid metabolic stress (S100A6), MYC-driven fatty acid biosynthesis (pMYC), and proliferative activation (Ki67). Per-core mean Metabolic-EMT scores were compared between SP and SR phenotypes using the Wilcoxon rank-sum test. Spatial pseudo-images were generated by plotting single-cell coordinates colored by annotated cell type. All CyCIF analyses were performed in R (v4.3.2) using Seurat v5.0.1), tidyverse (v2.0.0), ggplot2 (v3.4.2), and ggrepel (v0.9.3).

### 4.8. Data Availability

All scRNA-seq data analyzed in this study are publicly available through GEO under accession number GSE176078 [13]. Bulk RNA-seq data are available under accession numbers GSE25066 [14] and GSE58812 [15]. The CyCIF publicly available data used in this study are available through GitHub [16] at https://github.com/engjen/MYC-PTENfl-mouse, accessed on 3 May 2026. All data generated in this study are provided in the Supplementary Dataset. The R code and analytical pipeline used in this study are publicly available at https://github.com/zdoha3234/TNBC-FA-EMT-Chemoresistance, accessed on 21 June 2026.

## 5. Conclusions

This study identifies a Fatty Acid–EMT (FA-EMT) epithelial subset as a master driver of chemotherapy resistance in TNBC that actively reprograms the tumor microenvironment through coordinated MDK–NCL and MIF–CD74–CXCR4 ligand–receptor signaling, simultaneously suppressing adaptive and innate anti-tumor immunity. Integrating single-cell resolution, independent bulk transcriptomic validation across 277 TNBC patients, and spatial CyCIF validation in a published TNBC mouse model, we demonstrate that FA-EMT co-expression—rather than either program individually—most powerfully predicts chemotherapy resistance and reduced overall survival, and that FA-EMT cells physically co-localize with immunosuppressive MDSC and Treg populations in resistant tumor tissue, establishing this subset as a functionally integrated resistance mechanism and a spatially confirmed clinically actionable biomarker. MDK–NCL and MIF–CD74–CXCR4 represent mechanistically grounded therapeutic targets whose disruption could simultaneously dismantle this immunosuppressive niche and sensitize chemotherapy-resistant TNBC to cytotoxic treatment. Clinical investigation of these axes in combination with neoadjuvant chemotherapy—stratified by FA-EMT co-expression scoring—represents the critical next step toward improving pathological complete response rates and long-term survival in this aggressive disease.

## Data Availability

All scRNA-seq data analyzed in this study are publicly available through the Gene Expression Omnibus (GEO) under accession number GSE176078 [13]. Bulk transcriptomic validation data are available under accession numbers GSE25066 [14] and GSE58812 [15]. The CyCIF publicly available data used in this study are available on GitHub [16] at https://github.com/engjen/MYC-PTENfl-mouse, accessed on 3 May 2026. All supplementary data generated in this study, including differential gene expression tables and ligand–receptor interaction datasets, are provided in the Supplementary Dataset (Tables S1–S11). The R code and complete analytical pipeline used in this study are publicly available at https://github.com/zdoha3234/TNBC-FA-EMT-Chemoresistance, accessed on 21 June 2026.

## Supplementary Materials

The following supporting information can be downloaded at: https://www.mdpi.com/article/10.3390/ijms27146157/s1.
