# Supplementary material for: Integrative Single-Cell and Bulk Transcriptomic Analyses with Spatial Validation Identify a Residual Fatty Acid–EMT Subset Driving Chemotherapy Resistance in Triple-Negative Breast Cancer via MIF- and MK-Mediated Ligand–Receptor Signaling
Source: Int J Mol Sci. 2026 Jul 9;27(14):6157. doi: 10.3390/ijms27146157 (PMC13409733; doi:10.3390/ijms27146157)
Supplement: Supplementary file 1 [file ijms-27-06157-s001.zip › Supplementry_Figure.pdf]

SUPPLEMENTARY FIGURES

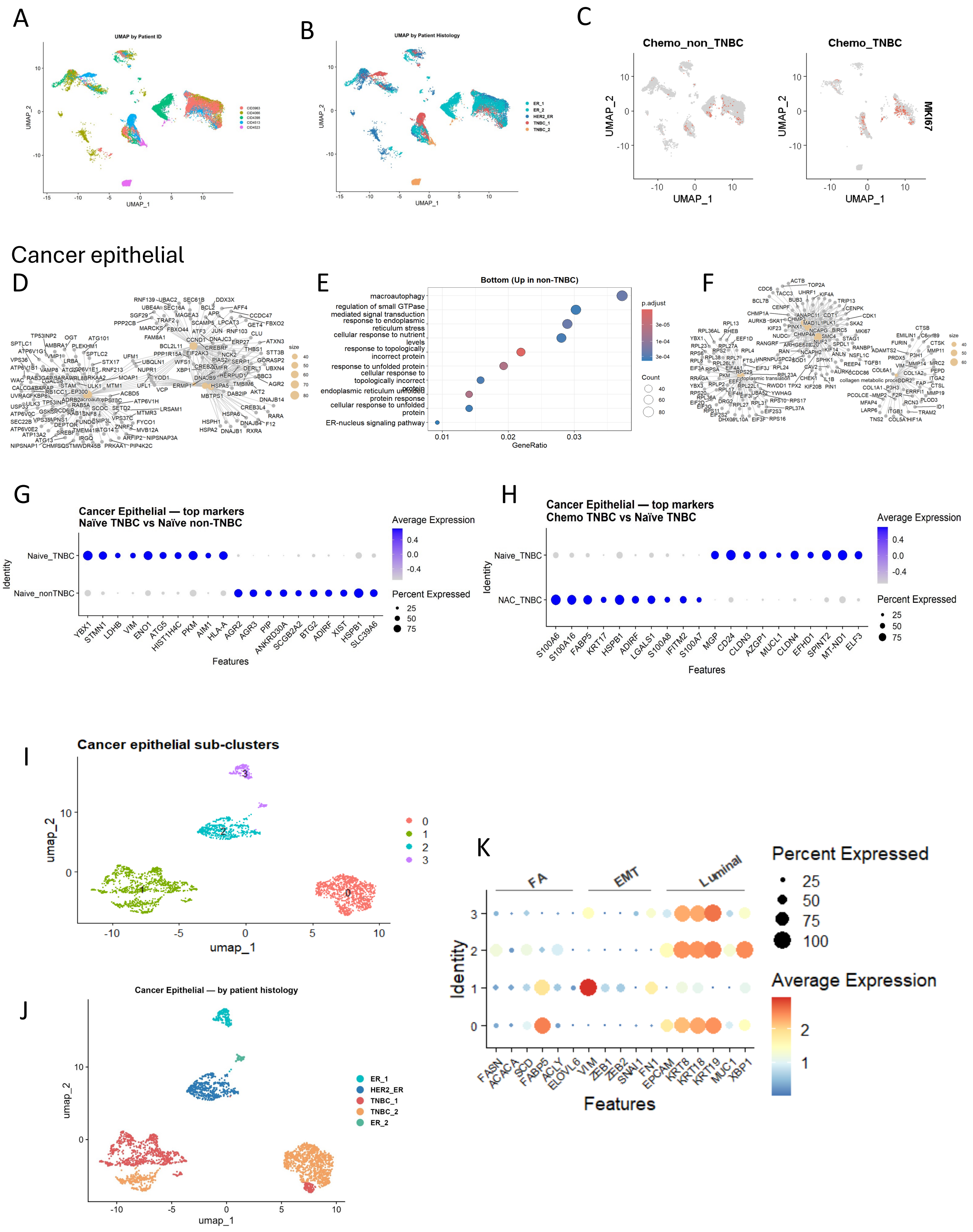

Supplementary Figure S1. Single-cell transcriptomic characterization of the Wu et al. 2021 breast cancer atlas and cancer epithelial subclusters. (A) UMAP projection of all profiled cells colored by patient ID (CID prefix), illustrating the distribution of individual donors across the full atlas. (B) UMAP projection of all profiled cells colored by patient histological subtype (ER_1, ER_2, HER2_ER, TNBC_1, TNBC_2), showing subtype-level transcriptional organization. (C) Split UMAP feature plots showing MKI67 expression overlaid on cancer epithelial cells in

Chemo_non_TNBC (left) and Chemo_TNBC (right) groups. (D) Gene network (cnet) plot illustrating the connectivity of significantly upregulated genes in Chemo_TNBC cancer epithelial cells and their associated enriched biological process terms. (E) Dot plot of Gene Ontology (GO) biological process enrichment for genes upregulated in Chemo_non_TNBC cancer epithelial cells (Benjamini–Hochberg correction). Dot size: gene count per term; color: adjusted p-value. (F) Gene network (cnet) plot illustrating the connectivity of significantly upregulated genes in Chemo_non_TNBC cancer epithelial cells and their associated enriched biological process terms. (G) Dot plot showing average expression and percent expression of top marker genes in cancer epithelial cells from differentially expressed marker genes in Naïve_TNBC versus Naïve_non_TNBC (Analysis validation1;n = 8,389 vs 13,132 cells). (H) Dot plot showing average expression and percent expression of top marker genes in cancer epithelial cells from differentially expressed marker genes in Chemo_TNBC versus Naïve_TNBC (Analysis validation2; n = 2,225 vs 8,389 cells). (I) UMAP of cancer epithelial subclusters (clusters 0–3) derived from unsupervised of cancer epithelial cells from the Chemo-treated cohort (4 patients). (J) UMAP of cancer epithelial subclusters colored by patient histology (ER_1, ER_2, HER2_ER, TNBC_1, TNBC_2), illustrating the patient-of-origin distribution across subclusters. (K) Dot plot of FA (fatty acid), EMT, and Luminal signature marker genes across cancer epithelial subclusters (0–3), showing average expression and percent of cells expressing each gene.

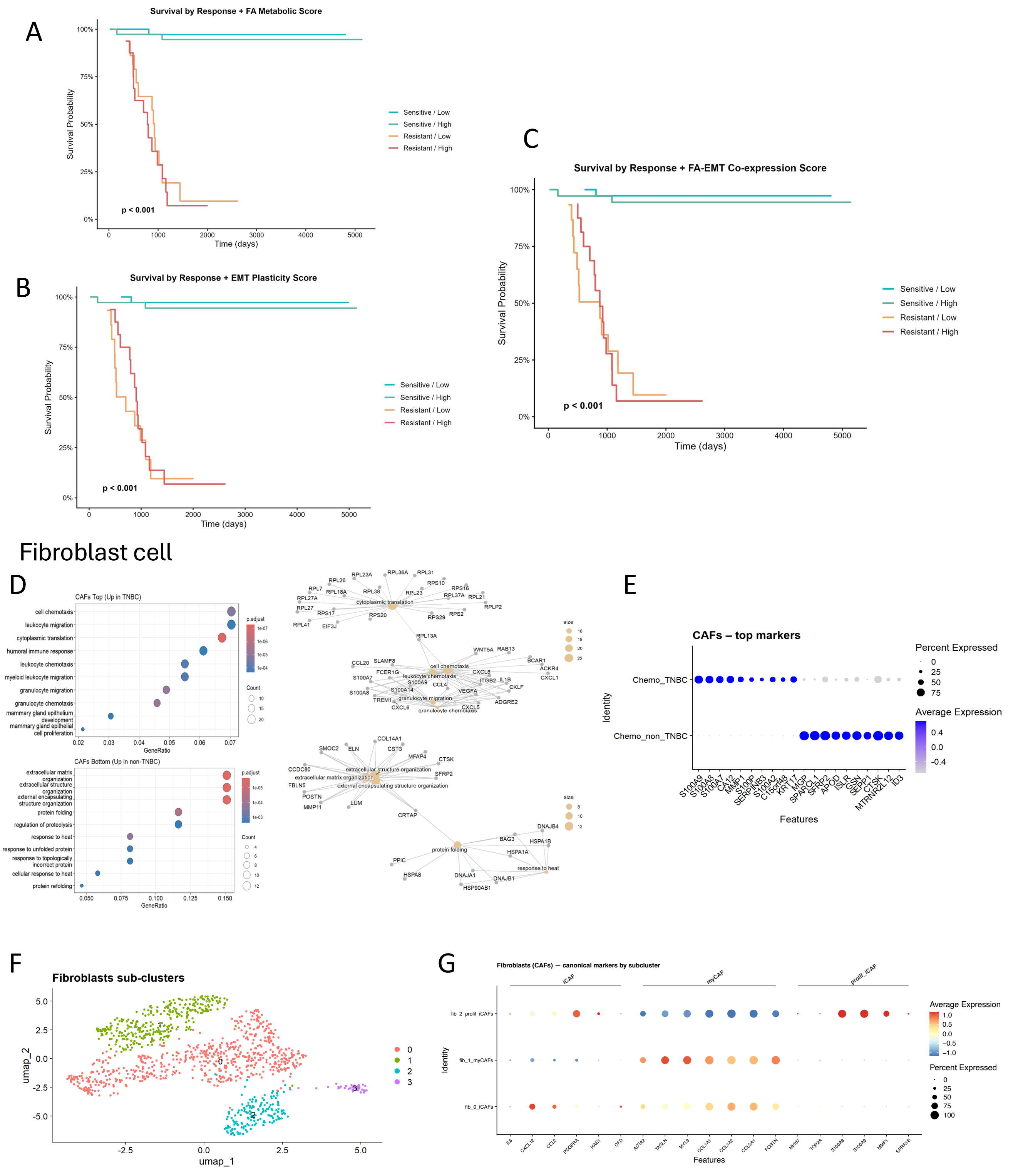

Supplementary Figure S2. Canonical marker expression and sub-cluster characterization of the T-cell and myeloid compartments. T-cell compartment: A–C: Bulk transcriptomic cohort (GSE25066) (A) Kaplan–Meier survival curves stratified by chemotherapy response (Sensitive/Resistant) and FA Metabolic score (Low/High), yielding four groups. Log-rank p < 0.001. (B) Kaplan–Meier survival curves stratified by chemotherapy response and EMT Plasticity score (Low/High). Log-rank p < 0.001. (C) Kaplan–Meier survival curves stratified by chemotherapy response and FA-EMT co-expression score (Low/High). Log-rank p < 0.001. D**–G: Cancer-associated fibroblast (CAF) compartment (scRNA-seq); (D)** Top left: Dot plot of gene ontology (GO) biological process enrichment for genes upregulated in Chemo_TNBC CAFs (Benjamini–Hochberg correction). Dot size: gene count per term; color: adjusted *p*-value. Top right: Gene network (cnet) plot illustrating the connectivity of

significantly upregulated genes in Chemo_TNBC CAF cells. Bottom left: Dot plot of GO biological process enrichment for genes upregulated in Chemo_non_TNBC CAFs (Benjamini–Hochberg correction). Bottom right: Gene network (cnet) plot illustrating the connectivity of significantly upregulated genes in Chemo_non_TNBC CAF cells. **(E)** Dot plot of top differentially expressed marker genes across chemotherapy groups in CAFs. Dot size: percentage of expressing cells; color intensity: scaled mean expression (blue, high; grey, low). **(F)** Annotated sub-cluster UMAP of the fibroblast (CAF) compartment identifying three subtypes: iCAFs, myCAFs, and prolif_iCAFs. **(G)** Dot plot of canonical marker gene expression across fibroblast sub-clusters (iCAFs, myCAFs, prolif_iCAFs). Marker groups: iCAF markers (*IL6*, *CXCL12*, *CCL2*, *PDGFRA*), myCAF markers (*ACTA2*, *TAGLN*, *COL1A1*, *POSTN*), and prolif_iCAF markers (*MKI67*, *S100A8*, *MMP1*). Dot size: percentage of expressing cells; color intensity: scaled mean expression.

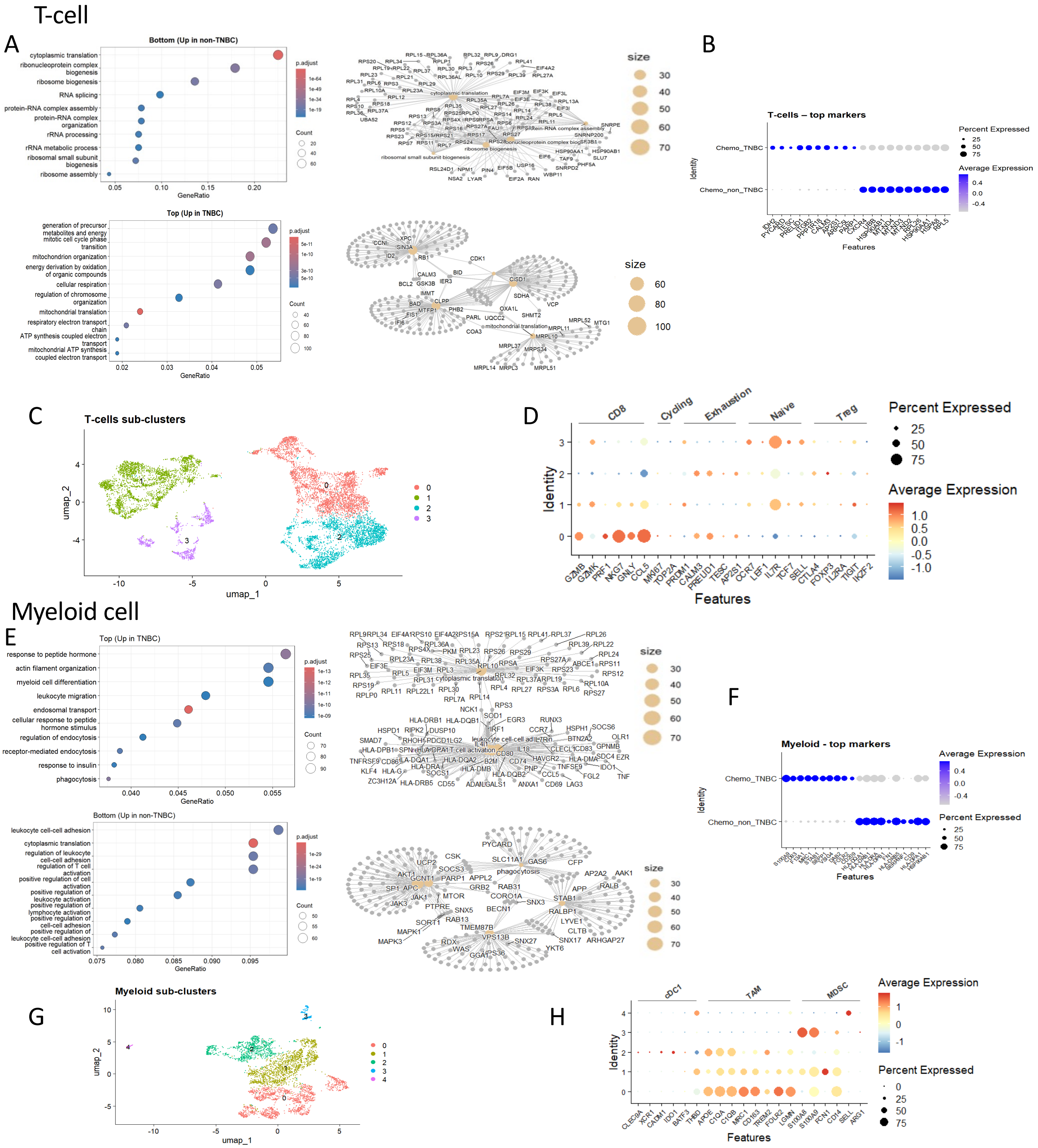

Supplementary Figure S3. Canonical marker expression and sub-cluster characterization of the T-cell and myeloid compartments. T-cell compartment: (A) Top left: Dot plot of gene ontology (GO) biological process enrichment for genes upregulated in Chemo_TNBC T cells (Benjamini–Hochberg correction). Dot size: gene count per term; color: adjusted *p*-value. Top right: Gene network (cnet) plot illustrating the connectivity of significantly upregulated genes in Chemo_TNBC T cells. Bottom left: Dot plot of GO biological process enrichment for genes upregulated in Chemo_non_TNBC T cells (Benjamini–Hochberg correction). Bottom right: Gene network (cnet) plot illustrating the connectivity of significantly upregulated genes in Chemo_non_TNBC T cells. **(B)** Dot plot of top differentially expressed marker genes across chemotherapy groups in T cells. Dot

size: percentage of expressing cells; color intensity: scaled mean expression (blue, high; grey, low). **(C)** Annotated sub-cluster UMAP of the T-cell compartment identifying three subtypes: CD8_Exhausted, Naive_Memory, and Treg. **(D)** Dot plot of canonical marker gene expression across T-cell sub-clusters (CD8_Exhausted, Naive_Memory, Treg). Marker groups: Cycling (*MKI67*, *TOP2A*), Exhaustion (*PRDM1*, *CALM3*, *PRELID1*, *TESC*, *AP2S1*), γδ T cells (*TRGC2*, *TRDV2*), Naive (*CCR7*, *LEF1*, *TCF7*, *SELL*, *IL7R*), Treg (*FOXP3*, *CTLA4*, *IL2RA*, *TIGIT*, *IKZF2*), and Cytotoxic (*GZMB*, *GZMK*, *PRF1*, *NKG7*, *GNLY*, *CCL5*). Dot size: percentage of expressing cells; color intensity: scaled mean expression. **Myeloid compartment: (E)** Top left: Dot plot of gene ontology (GO) biological process enrichment for genes upregulated in Chemo_TNBC myeloid cells (Benjamini–Hochberg correction). Dot size: gene count per term; color: adjusted *p*-value. Top right: Gene network (cnet) plot illustrating the connectivity of significantly upregulated genes in Chemo_TNBC myeloid cells. Bottom left: Dot plot of GO biological process enrichment for genes upregulated in Chemo_non_TNBC myeloid cells (Benjamini–Hochberg correction). Bottom right: Gene network (cnet) plot illustrating the connectivity of significantly upregulated genes in Chemo_non_TNBC myeloid cells. **(F)** Dot plot of top differentially expressed marker genes across chemotherapy groups in myeloid cells. Dot size: percentage of expressing cells; color intensity: scaled mean expression (blue, high; grey, low). **(G)** Annotated sub-cluster UMAP of the myeloid compartment identifying three subtypes: TAMs, cDC1, and MDSCs. **(H)** Dot plot of canonical marker gene expression across myeloid sub-clusters (TAMs, cDC1, MDSCs). Marker groups: cDC1 (*CLEC9A*, *XCR1*, *CADM1*, *IDO1*, *BATF3*, *THBD*), TAM (*APOE*, *C1QA*, *C1QB*, *MRC1*, *CD163*, *TREM2*, *FOLR2*, *LGMN*), and MDSC (*S100A8*, *S100A9*, *FCN1*, *CD14*, *SELL*, *ARG1*). Dot size: percentage of expressing cells; color intensity: scaled mean expression.

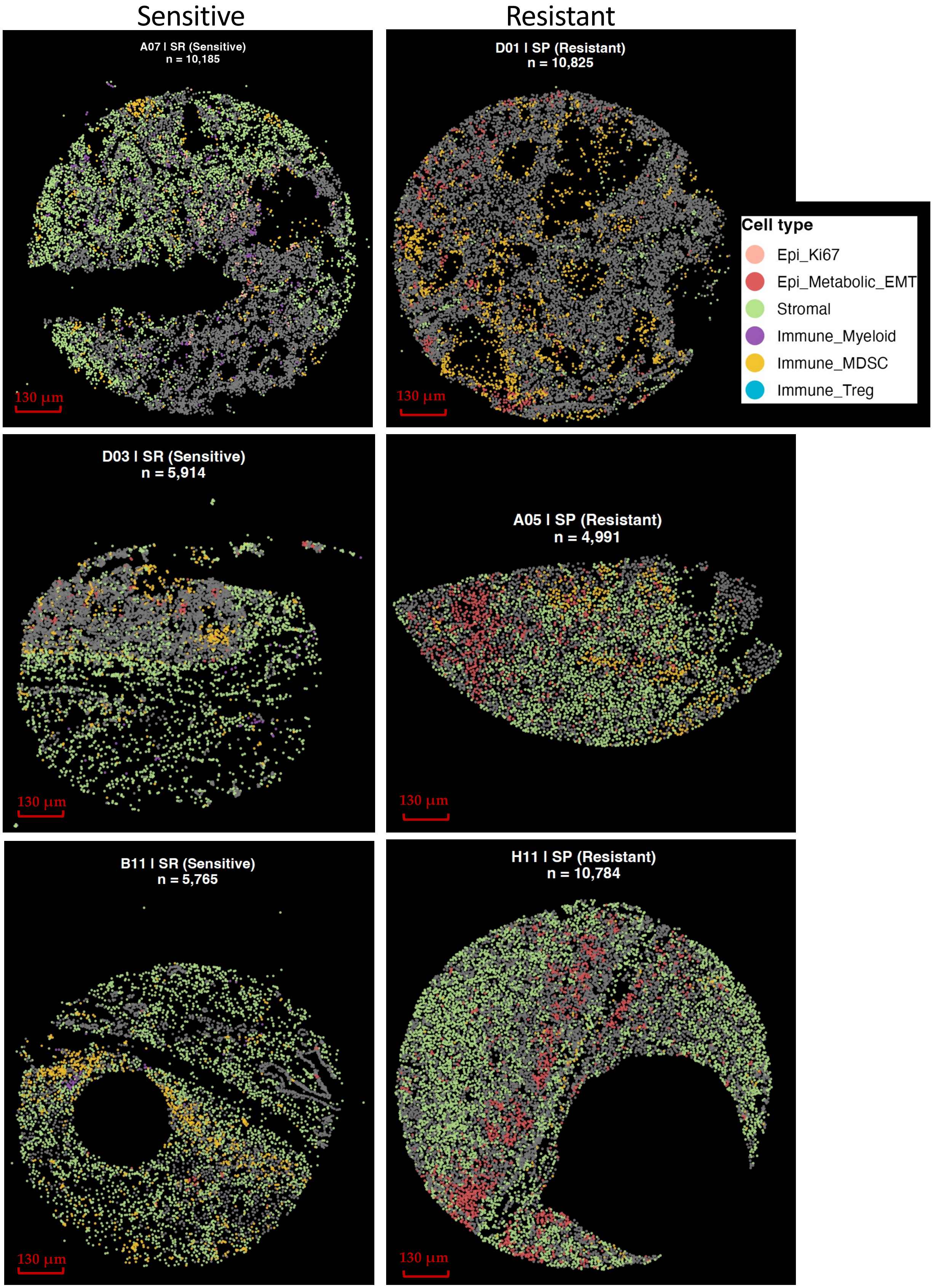

Supplementary Figure S4. CyCIF spatial pseudo-images of additional SP (Resistant) and SR (Sensitive) tumor cores. Spatial pseudo-images of three SR sensitive cores (on the left) (A07, n = 10,185; D03, n = 5,914; B11, n = 5,765) and three SP resistant cores (on the right) (D01, n = 10,825; A05, n = 4,991; H11, n = 10,784), selected to represent the full range of core sizes across the TMA. Each dot represents a single profiled cell, colored by assigned cell type: Epi_Ki67 (light pink), Epi_Metabolic_EMT (red), Stromal (green), Immune_Myeloid (purple), Immune_MDSC (orange), and Immune_Treg (teal).
